# Discovery of indazole-carboxamide based USP8 inhibitor for immune activation and antitumor response in colorectal cancer

**DOI:** 10.1016/j.tranon.2026.102956

**Published:** 2026-08-05

**Authors:** Zhiyu Song, Yuanying Wang, Weiwei Zhou, Xiaomei Huang, Gang Jia, Mingyang Han

**Affiliations:** a Department of Pharmacy, Henan Provincial People's Hospital; People's Hospital of Zhengzhou University, Zhengzhou, Henan, 450003, China; b People's Hospital of Zhengzhou University, Zhengzhou, Henan, 450003, China; c Department of oncology, Henan Provincial People's Hospital;People's Hospital of Zhengzhou University, Zhengzhou, Henan, 450003, China; d Department of Gastrointestinal Surgery, Henan Provincial People's Hospital; People's Hospital of Zhengzhou University, Zhengzhou, Henan, 450003, China

**Keywords:** Colorectal cancer, USP8, PD-L1, CD8⁺ T cells, Immune evasion, Deubiquitinase inhibitor

## Abstract

**Background:**

Microsatellite-stable colorectal cancer (CRC), which accounts for most CRC cases, responds poorly to immune checkpoint inhibitors. Increasing evidence suggests that the deubiquitinase USP8 maintains PD-L1 protein expression by erasing its K48-linked ubiquitination, thereby preserving membrane PD-L1 and facilitating immune escape. How pharmacological inhibition of USP8 regulates PD-L1 stability and antitumor immunity in CRC, however, has not been fully clarified.

**Methods:**

Through structure-based optimization, we developed a novel indazole-carboxamide based compound, W-17, as a potent USP8 inhibitor. The effects of W-17 on PD-L1 stability, CD8⁺ T-cell cytotoxicity, and tumor growth were evaluated using enzymatic assays, cellular thermal shift assays (CETSA), Western blotting, T-cell killing assays as well as a syngeneic CT26 CRC mouse model.

**Results:**

W-17 inhibited USP8 enzymatic activity (IC_50_ = 83 nM), promoted PD-L1 polyubiquitination, and induced its proteasomal degradation. Pharmacological blockade of USP8 enhanced CD8⁺ T-cell mediated killing and increased the secretion of interferon-γ (IFN-γ) and tumor necrosis factor-α (TNF-α) *in vitro*. In an immunocompetent CT26 mouse model, W-17 significantly suppressed tumor growth, increased intratumoral CD8⁺ T-cell infiltration, and reduced PD-L1 expression, without overt systemic toxicity.

**Conclusion:**

These findings identify USP8 as a post-translational regulator of PD-L1 stability in colorectal cancer and demonstrate that the novel USP8 inhibitor W-17 effectively promotes PD-L1 degradation and restores CD8⁺ T-cell-mediated antitumor immunity. Our study highlights W-17 as a promising lead compound for the development of USP8-targeted immunotherapeutic strategies for microsatellite-stable colorectal cancer.

## Highlights

• W‑17 identified as a potent, selective USP8 inhibitor with sub‑100 nM activity.
• W‑17 induces K48 ubiquitination and proteasomal degradation of PD‑L1 post‑translationally.
• USP8 inhibition boosts CD8⁺ T‑cell infiltration and antitumor cytotoxic responses.
• W‑17 delivers over 70% tumor suppression in CT26 models without systemic toxicity.

## Introduction

Colorectal cancer (CRC) remains one of the most prevalent malignancies all over the world and ranks as a leading cause of cancer-related mortality, with more than 1.9 million new cases and over 935,000 deaths reported in 2022 according to GLOBOCAN [1]. Although early-stage disease can often be managed surgically or with standardized chemotherapeutic regimens, outcomes for patients with advanced CRC remain unsatisfactory, the 5-year survival rate of metastatic CRC is still below 15% [2]. Most CRC cases are microsatellite-stable (MSS) tumors, which are notably resistant to immune targeting therapeutics such as PD-1 and PD-L1 antibodies [3,4]. This immune-refractory phenotype represents a major obstacle to durable clinical benefit from current immunotherapies.

Tumor immune evasion is largely driven by the PD-1/PD-L1 immune checkpoint axis, which suppresses cytotoxic T-cell activity and promotes T-cell exhaustion [5]. Elevated PD-L1 expression on tumor cells correlates with advanced stage, poor prognosis, and resistance to T cell-mediated killing [6]. Although transcriptional regulation of PD-L1 by oncogenic signaling pathways (e.g., STAT3, MYC, PI3K/AKT) has been well documented [7], emerging studies highlight that PD-L1 protein stability is predominantly controlled by post-translational modifications, especially ubiquitination and deubiquitination [[8], [9], [10]]. The dynamic ubiquitin–proteasome system dictates PD-L1 turnover and surface abundance, directly shaping tumor immune evasion [11].

Among the deubiquitinases (DUBs), USP8 (also known as UBPY) has recently emerged as a pivotal regulator of PD-L1 stability in solid tumors [12]. USP8 removes ubiquitin chains from PD-L1, thereby preventing its proteasome-mediated degradation and sustaining its membrane localization [12]. High USP8 expression has been reported in colorectal, gastric, and breast cancers and correlates with aggressive phenotypes, immunosuppressive tumor microenvironments, and poor patient survival [[13], [14], [15], [16]]. Genetic silencing or pharmacological inhibition of USP8 markedly reduces PD-L1 expression, enhances CD8⁺ T-cell infiltration, and restores antitumor immunity in preclinical models [12]. These findings position USP8 as a crucial post-translational regulator of PD-L1 and a promising immunotherapeutic target.

Despite its emerging relevance, selective pharmacological inhibitors of USP8 remain scarce. Most previous DUB-targeted strategies have focused on USP7, USP22 and so on [[17], [18], [19], [20], [21]], while USP8 inhibition has only recently attracted attention. Several small-molecule USP8 inhibitors, including DUBs-IN-2 and FDL-08, have shown preclinical activity but remain in early discovery stages and have not been evaluated in CRC [22,23]. Therefore, developing potent and selective USP8 inhibitors represents a compelling therapeutic avenue to overcome immune resistance in MSS CRC.

In this study, we identified and optimized W-17, a novel o-aminodiphenyl-based USP8 inhibitor, through structure-guided design. We demonstrate that W-17 potently inhibits USP8 enzymatic activity, promotes PD-L1 K48-lined ubiquitination and proteasomal degradation, enhances CD8⁺ T-cell cytotoxicity, and suppresses tumor growth in a syngeneic CRC model. These findings give a mechanistic rationale and preclinical foundation for targeting USP8 to restore antitumor immune responses in microsatellite-stable colorectal cancer.

## Result

### USP8 is abundantly expressed in CRC and associates with immune-evasive features

**Fig. 1:**
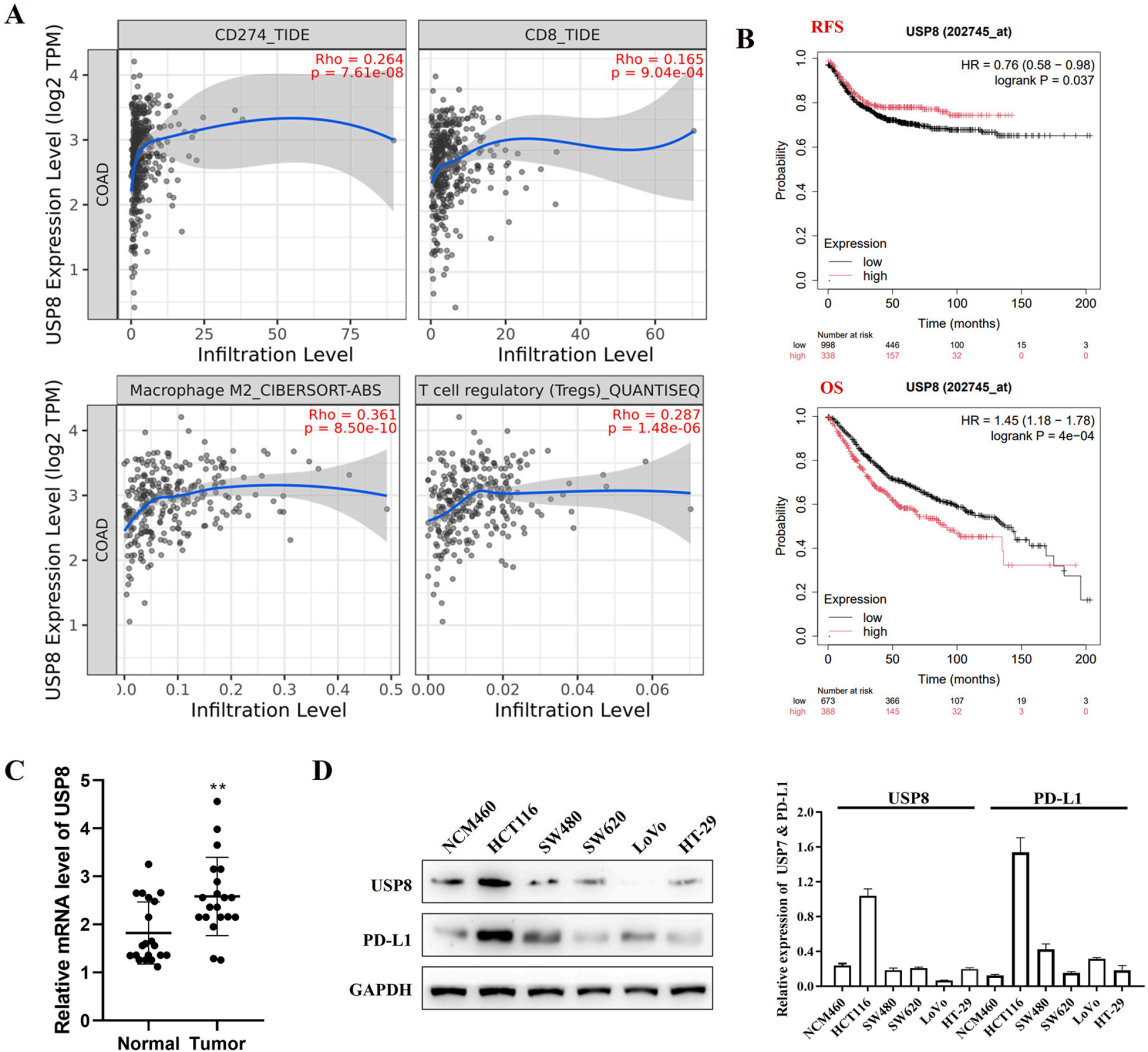
USP8 expression landscape and immune features in CRC. (A) Purity-adjusted analyses in TIMER3.0 reveal that high USP8 mRNA level correlates with T-cell dysfunction/exhaustion signatures (n = 456); (B) Kaplan-Meier survival analysis shows the correlation between USP8 expression and OS and RFS; (C) qPCR analysis showing USP8 mRNA expression levels in tumor and adjacent normal tissues from colorectal cancer patients (n = 20); (D) Western blot analysis about the expressions of USP8 and PD-L1 in indicated cell lines. Data were presented as mean ± SD.

To understand the immune context, TIMER3.0 was applied to evaluate the association between USP8 mRNA level and immune-related features in colorectal cancer. Purity-adjusted Spearman correlations were calculated. The level of USP8 mRNA positively correlated with TIDE-derived CD274 (PD-L1) immune evasion scores and CD8⁺ T cell dysfunction scores, as well as increased M2 macrophage infiltration (CIBERSORT-ABS) and regulatory T cell abundance (quanTIseq), supporting a link between USP8 and an immunosuppressive tumor microenvironment (Fig. 1A) [24]. Survival meta-analysis using the colon subsystem of KM-plotter in Fig. 1B revealed that patients in the top USP8 quantile had improved recurrence-free survival (RFS) but poorer overall survival (OS) (log-rank p<0.05), which may be attributed to several factors, including differences in subsequent treatments after recurrence, tumor heterogeneity, and the complex role of USP8 in tumor progression and immune regulation [25]. Furthermore, paired tumor and adjacent normal tissues were obtained from 20 colorectal cancer patients. And our qPCR analysis in Fig. 1C also demonstrated that USP8 mRNA expression was significantly upregulated in tumor tissues compared with adjacent normal tissues, which is consistent with previous studies [16]. In cellular level, immunoblotting confirmed robust USP8 in HCT116, SW480, SW620, LoVo and HT29 relative to NCM460 (Fig. 1D). Notably, PD-L1 expression was also elevated in cell lines with high USP8 expression. Together, these data demonstrate that USP8 is linked to immunosuppressive features, thereby motivating the subsequent mechanistic and therapeutic studies, and are consistent with recent reports showing that targeting USP8 modulates PD-L1 biology and potentiates PD-L1 blockade.

### Identification and structure-guided optimization yield W-17 as a potent USP8 inhibitor

To identify novel USP8 inhibitors, we screened our in-house compound library comprising 2,591 diverse small molecule. This initial high-throughput screening campaign identified W-01 as a initial hit, exhibiting moderate USP8 inhibitory activity (IC_50_ = 3.073 µM).

We first investigated the structure-activity relationship (SAR) around the aryl group while retaining the thiomorpholine amide moiety. Substituting the pyridin-3-yl moiety in W-01 with an unsubstituted phenyl ring (W-02) resulted in a marked decrease in inhibitory activity (IC_50_ > 10 µM). Conversely, the pyrimidin-5-yl analog (W-03) maintained comparable activity (IC_50_ = 4.571 µM). A significant enhancement in activity was achieved by incorporating a 1-methyl-1H-pyrazol-4-yl group (W-04), which improved the IC_50_ approximately five-fold to 0.672 µM, identifying this heterocycle as a key pharmacophore for potency. Further modifications, including the exploration of other aromatic systems such as indole (W-06, W-07), 1H-pyrrolo [2,3-b]pyridine (W-08), and benzo [b]thiophene (W-09), yielded compounds with either maintained or diminished activity, confirming the optimal nature of the unsubstituted 1-methyl-1H-pyrazol-4-yl group for this position (Table 1).

**Table 1:** Anti-USP8 enzymatic activity of compounds WS-1∼W-09.

^a^ All experiments were performed independently at least three times, and the data are shown as the mean ± SD.

With the optimal 1-methyl-1H-pyrazol-4-yl group fixed, we focused on optimizing the amine portion (R^2^) to further enhance potency (Table 2). Replacement of the thiomorpholine in W-04 with piperazine (W-10) yielded a significant boost in activity (IC_50_ = 0.411 µM). Building on this, *N*-methylation of the piperazine gave compound W-11, which showed slightly reduced potency (IC_50_ = 0.588 µM). Its corresponding Boc-protected intermediate, W-12, exhibited further optimized activity (IC_50_ = 0.791 µM).

**Table 2:** Anti-USP8 enzymatic activity of compounds WS-10∼W-20.

^a^ All experiments were performed independently at least three times, and the data are shown as the mean ± SD.

Extension of the piperazine to a 4-(methylsulfonyl)phenylpiperazine (W-13) maintained strong inhibitory activity (IC_50_ = 0.337 µM). Potent inhibition was also observed with simple alicyclic amines, including morpholine (W-14) and piperidine (W-15), which displayed IC_50_ values of 0.251 µM and 0.811 µM, respectively. Replacing the amine with an N-methylpiperidin-4-amine structure gave W-16, which retained comparable activity (IC_50_ = 0.176 µM). A critical breakthrough was achieved by examining the oxidation state of the thiomorpholine sulfur. Oxidation of the thiomorpholine sulfide in W-04 to the corresponding sulfone gave compound W-17. This single modification dramatically enhanced inhibitory activity, resulting in W-17 with an IC_50_ of 0.135 µM —representing an approximately 5-fold improvement over the parent sulfide and positioning it as the most potent compound in the series. Other structural explorations, including pyrrolidine (W-18) and various spirocyclic diamines (W-19 and W-20) proved less effective. The simple anilide analog W-21 showed markedly reduced potency (IC_50_ = 2.111 µM), underscoring the importance of the basic amine moiety.

In summary, W-17 was prioritized as the lead USP8 inhibitor. Although W-16 exhibited comparable enzymatic potency, W-17 was favored for its less basic thiomorpholine-1,1-dioxide moiety, which was expected to reduce the risks of hERG inhibition and lysosomal trapping associated with the N-methylpiperidine group of W-16 [26,27]. Moreover, oxidation of W-04 to W-17 improved potency by approximately 5-fold, and molecular docking (Section 2.3) suggested favorable interactions with key USP8 residues. Collectively, these data supported the selection of W-17 for subsequent cellular and *in vivo* studies.

### Molecular docking and molecular dynamics (MD) analyses

**Fig. 2:**
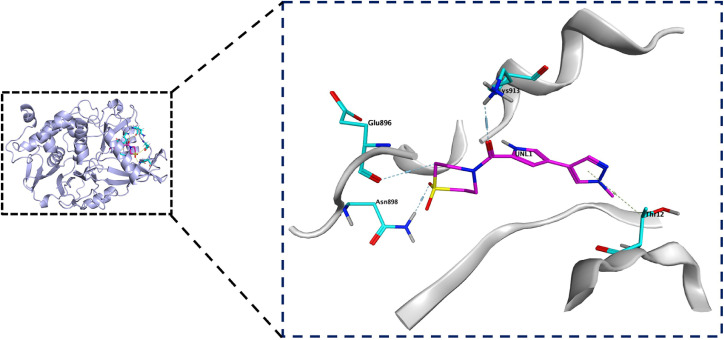
The proposed binding mode of **W-17** (Magenta sticks) in USP8 (PDB: 3N3K). Key residues are depicted, with hydrogen bonds and π-H bonds represented by blue and light green dotted lines, respectively.

The binding mode of W-17 with USP8 was investigated through Molecular Operating Environment (MOE). As shown in Fig. 2, W-17 effectively docked into the active binding pocket of USP8 (PDB code: 3N3K) [28] . The compound binds within the active site of USP8. Key interactions include hydrogen bonds between the ligand's carbonyl oxygen, thiomorpholine-1,1-dioxide oxygen, and methylene group and the side chains of Lys913, Asn898, and Glu896, respectively. Additionally, a π-H bond interaction is established between the pyrazole moiety and the backbone of Thr12.

**Fig. 3:**
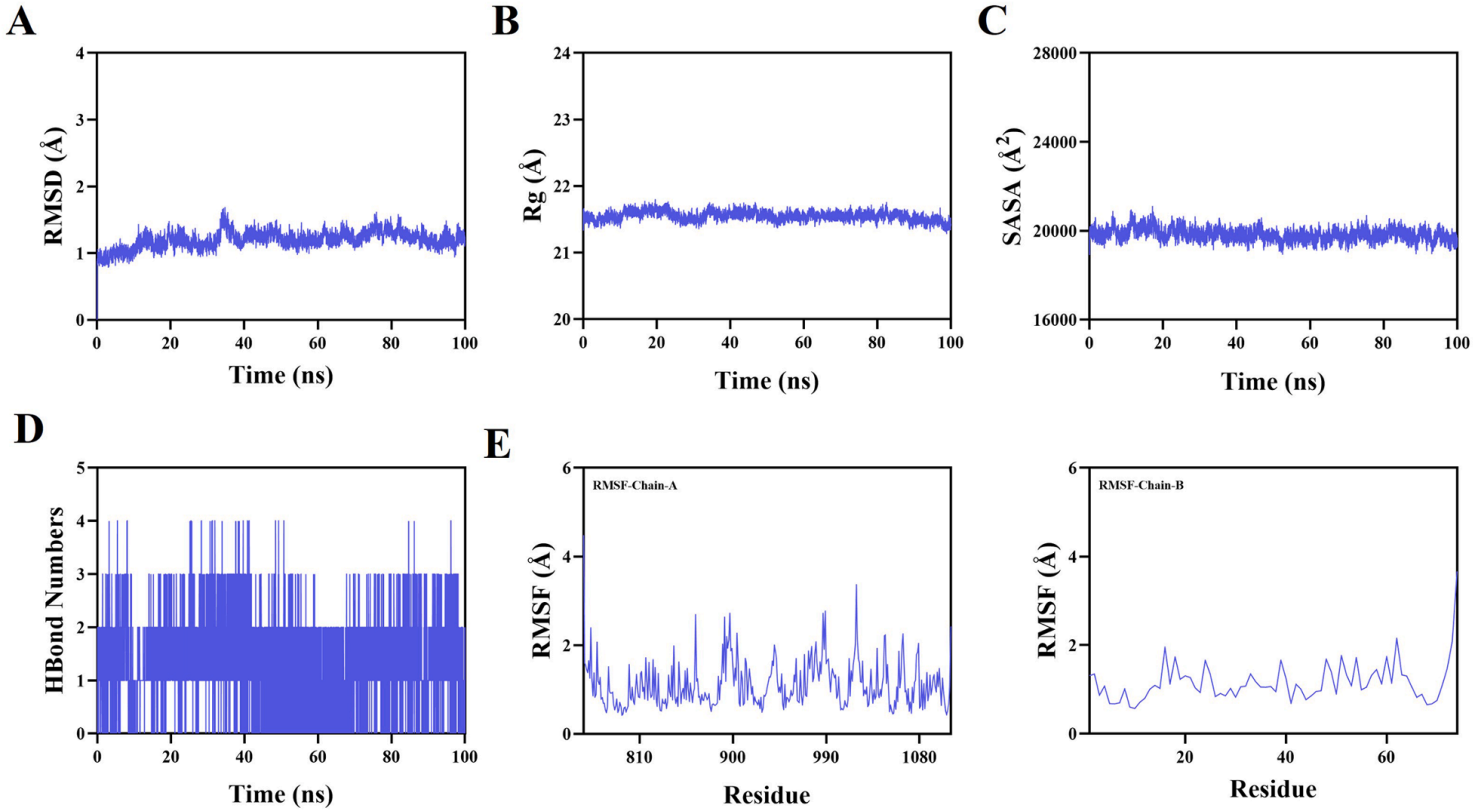
MD simulation analysis of the USP8-**W-17** complex. (A) RMSD; (B) Rg; (C) SASA; (D) Number of hydrogen bonds between the protein and ligand; (E) RMSF of USP8 residues.

To assess the dynamic stability of this interaction, a 100-ns MD simulation was performed. Analysis of the trajectory, presented in Fig. 3, confirmed the formation of a stable complex. Specifically, the root mean square deviation (RMSD) reached equilibrium after approximately 40 ns, exhibiting minor fluctuations around 1.3 Å (Fig. 3A), which points to a well-equilibrated binding mode. The global protein fold remained largely unaffected by ligand binding, as indicated by the consistent radius of gyration (Rg) (Fig. 3B) and the stable solvent-accessible surface area (SASA) (Fig. 3C). Hydrogen bond analysis revealed that W-17 maintains persistent interactions with USP8, forming approximately three hydrogen bonds for most of the simulation time (Fig. 3D), supporting stable ligand anchoring within the catalytic pocket. Furthermore, root mean square fluctuation (RMSF) analysis revealed constrained mobility for the majority of USP8 residues, particularly those lining the binding site (Fig. 3E), indicating local structural stabilization upon ligand engagement. Collectively, these results demonstrate that W-17 forms a conformationally stable complex with USP8, consistent with the proposed binding mode and its potent inhibitory activity.

### W-17 is a potent USP8 inhibitor *in vitro*

**Fig. 4:**
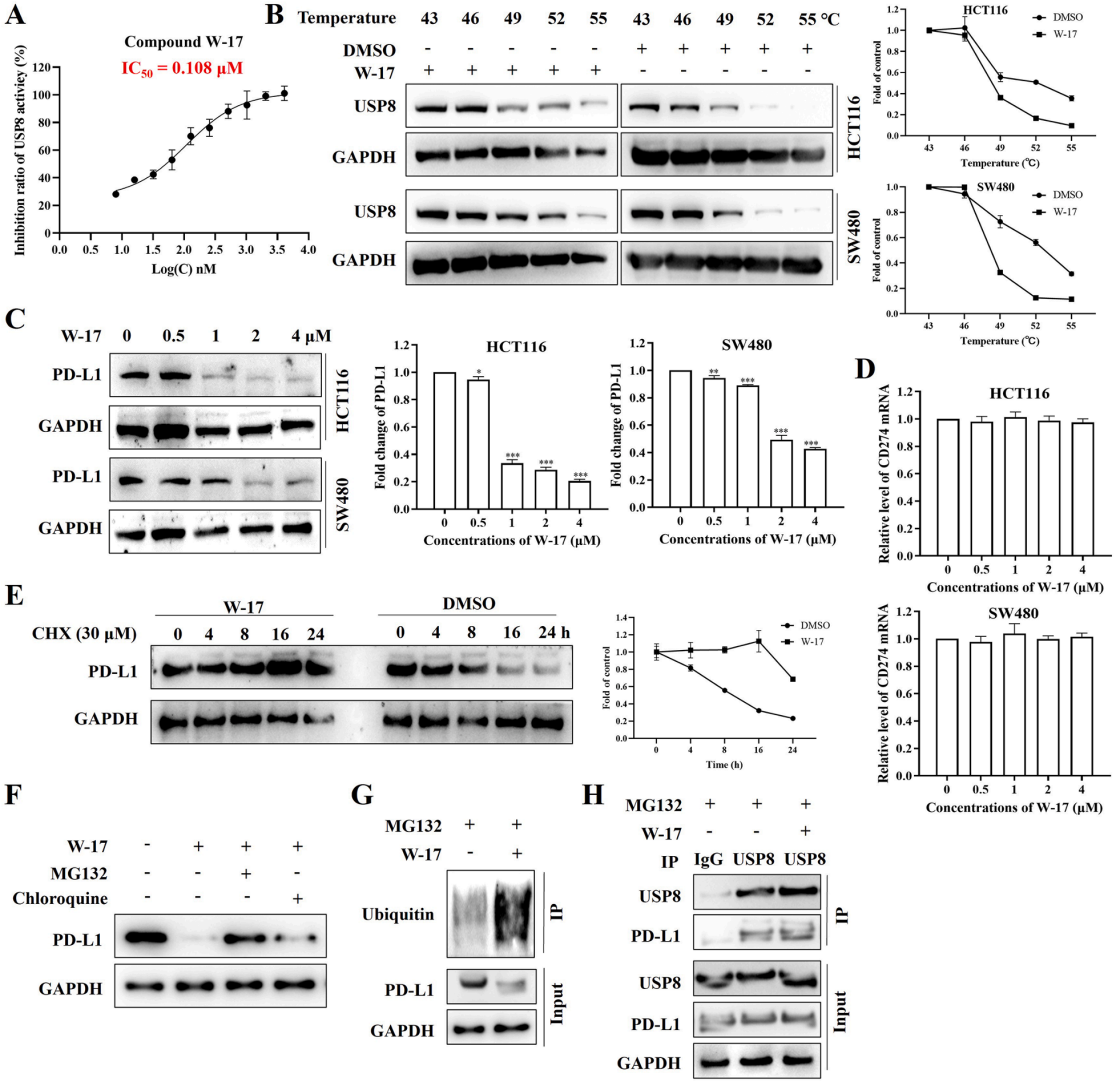
Compound **W-17** potently inhibits USP8 *in vitro*. (A) *In vitro* enzymatic assays establish W-17 as a potent and selective USP8 inhibitor; (B) CETSA assay demonstrate that W-17 stabilizes USP8 in HCT116 and SW480 cells; (C) Immunoblotting reveals a concentration dependent reduction of total PD-L1 protein; (D) CD274 mRNA remained unchanged following W-17 treatment; (E) Cycloheximide chase assays further show an obvious shortening of PD-L1 half-life; (F) Proteasome inhibition with MG132, but not lysosomal blockade, rescues W-17-induced PD-L1 loss; (G) W-17 treatment increased polyubiquitinated PD-L1; (H) Co-immunoprecipitation analyses show that W-17 does not disrupt the endogenous USP8/PD-L1 complex, consistent with catalytic inhibition rather than interference with substrate recognition. Data were presented as mean ± SD; *p < 0.05, **p < 0.01 and ***p < 0.001 was considered statistically significant.

Having established the potent *in vitro* inhibition of USP8 by W-17 (IC_50_ = 0.108 μM) (Fig. 4A), we sought to confirm its cellular target engagement with the aid of a cellular thermal shift assay (CETSA). It was found that W-17 can stabilize USP8 in HCT116 and SW480 cells, compared to the control group (Fig. 4B). To further evaluate the selectivity of W-17 toward USP8, we additionally examined its inhibitory activities against several representative USP family deubiquitinases, including USP7, USP10, and USP14. As shown in Table S1, W-17 exhibited minimal inhibitory effects on USP7, USP10, and USP14, with all IC₅₀ values greater than 10 μM. These results suggest that W-17 possesses relatively favorable selectivity toward USP8 within the tested USP family members.

Functionally, immunoblotting showed a concentration- and time-dependent reduction of total PD-L1 after W-17 exposure while CD274 mRNA remained unchanged, and cycloheximide chase demonstrated an obvious shortening of PD-L1 half-life, together indicating post-transcriptional control (Fig. 4C-E). Mechanistically, proteasome inhibition with MG132, but not lysosome blockade, rescued W-17 induced PD-L1 loss, and PD-L1 immunoprecipitation followed by linkage-specific ubiquitin blots revealed a marked increase in K48-linked polyubiquitination (with minimal change in K63), confirmed under denaturing IP conditions to reside on PD-L1 itself (Fig. 4F-G). Importantly, W-17 did not disrupt the endogenous USP8-PD-L1 complex, consistent with catalytic inhibition rather than interference with substrate recognition (Fig. 4H). Collectively, these data demonstrate that W-17 directly engages USP8 in cells and accelerates PD-L1 proteasomal degradation via enhanced K48-linked polyubiquitination, thereby providing the mechanistic basis for downstream immune-functional effects.

### W-17 reduces PD-L1 stability and enhances CD8⁺ T-cell mediated cytotoxicity

**Fig. 5:**
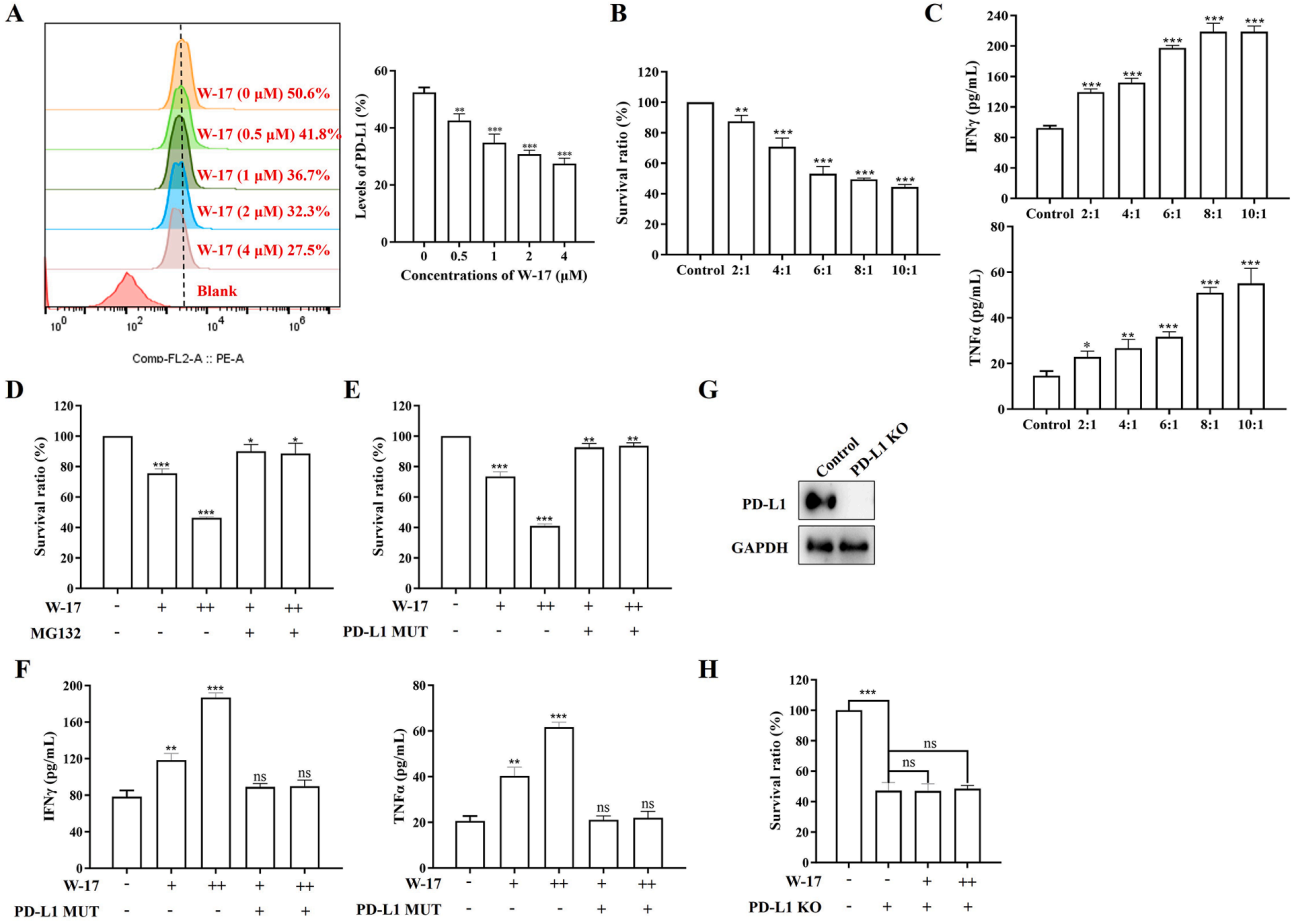
Compound W-17 can promote the destabilization of PD-L1 and enhance T-cell mediated tumor cell killing activity. (A) Flow cytometric analysis shows that W-17 pretreatment reduces surface PD-L1 expression on HCT-116 cells in a concentration-dependent manner; (B) W-17 significantly increases target-cell lysis relative to control, shifting the killing curves upward at all ratios tested; (C) Quantification of IFN-γ and TNF-α using Elisa in co-culture supernatants reveals elevated cytokine production upon W-17 treatment; (D) Proteasome blockade with MG132 attenuated the cytotoxicity enhancement induced by W-17, indicating proteasome-dependent pathway engagement; (E-F) Overexpression of PD-L1 triple mutant (K263R/K270R/K280R) renders tumor cells refractory to W-17 in both killing and cytokine readouts; (G) Western blot confirmed the genetic deletion of PD-L1 in HCT-116 cells; (H) Under PD-L1-deficient condition, W-17 confers no additional enhancement, confirming on-pathway action through the PD-L1 axis. Data were presented as mean ± SD; *p < 0.05, **p < 0.01 and ***p < 0.001 was considered statistically significant.

Building on the mechanistic finding that W-17 destabilizes PD-L1, we quantified functional consequences in tumor-T-cell co-cultures. Flow cytometry and related quantitative analysis showed that membrane PD-L1 levels decreased significantly after treatment with W-17 at concentrations of 0.5–4 μM, confirming that USP8 inhibition effectively reduces cell surface PD-L1 expression (Fig. 5A). When pretreated tumor cells were co-cultured with activated human CD8⁺ T cells across E:T ratios (1:1-10:1), W-17 significantly increased target-cell lysis relative to control, shifting the killing curve upward at all ratios tested (Fig. 5B). To link cytolysis with effector activation, we measured IFN-γ and TNF-α in the same supernatants by ELISA and observed higher cytokine levels with W-17, as shown in Fig. 5C. To further investigate whether the immunomodulatory effects of W-17 were dependent on USP8, we evaluated the activity of W-17 in USP8-knockout colorectal cancer cells. As shown in Figure S1, USP8 deficiency markedly attenuated the ability of W-17 to reduce PD-L1 expression. Consistently, the enhanced CD8⁺ T-cell-mediated cytotoxicity induced by W-17 was largely diminished in USP8-knockout cells, accompanied by reduced IFN-γ and TNF-α secretion in the co-culture system. These findings support that the immune regulatory effects of W-17 are, at least in part, mediated through USP8-dependent PD-L1 regulation.

To further investigate whether W-17 influences immune-related tumor cell death and antigen presentation pathways, we performed additional mechanistic studies. As shown in Figure S2, W-17 treatment increased calreticulin (CRT) exposure on the cell surface and promoted HMGB1 release in gastric cancer cells, suggesting the induction of immunogenic cell death (ICD)-associated features. In addition, W-17 significantly upregulated the expression of several MHC-I antigen presentation-related molecules, including HLA-A, HLA-B, HLA-C, and PSMB8, while no obvious changes were observed in TAP1, TAP2, or PSMB9 (Figure S3A). Western blot analysis further showed increased phosphorylation of p65 following W-17 treatment (Figure S3B), suggesting activation of NF-κB-associated immune signaling pathways [29]. These findings indicate that, in addition to PD-L1 downregulation, W-17 may further enhance CD8⁺ T-cell-associated antitumor immunity through modulation of IFN/MHC-I-related antigen presentation pathways and immune-relevant tumor cell death.

To verify pathway dependence, we blocked the proteasome with MG132, which restored PD-L1 abundance, proportionally attenuated the cytotoxicity increase produced by W-17, as shown in Fig. 5D. To further validate the substrate requirement, overexpression of a ubiquitination resistant PD-L1 triple mutant at K263R/K270R/K280R rendered tumor cells refractory to W-17 in both killing and cytokine readouts, as shown in Figs. 5E-F. To ensure that the effect tracks on the PD-L1 axis, deletion of PD-L1 elevated baseline T cell cytotoxicity in the CCK-8 assay and in this PD-L1-deleted background W-17 provided no additional improvement (Figs. 5G-H), which confirms on pathway action. Collectively, these data demonstrate that reducing tumor PD-L1 via USP8 inhibition directly augments CD8⁺ T-cell cytotoxicity and effector function in vitro, motivating in vivo evaluation of monotherapy and combination strategies.

### *In vivo* antitumor effects of the USP8 Inhibitor W-17 in colorectal cancer

**Fig. 6:**
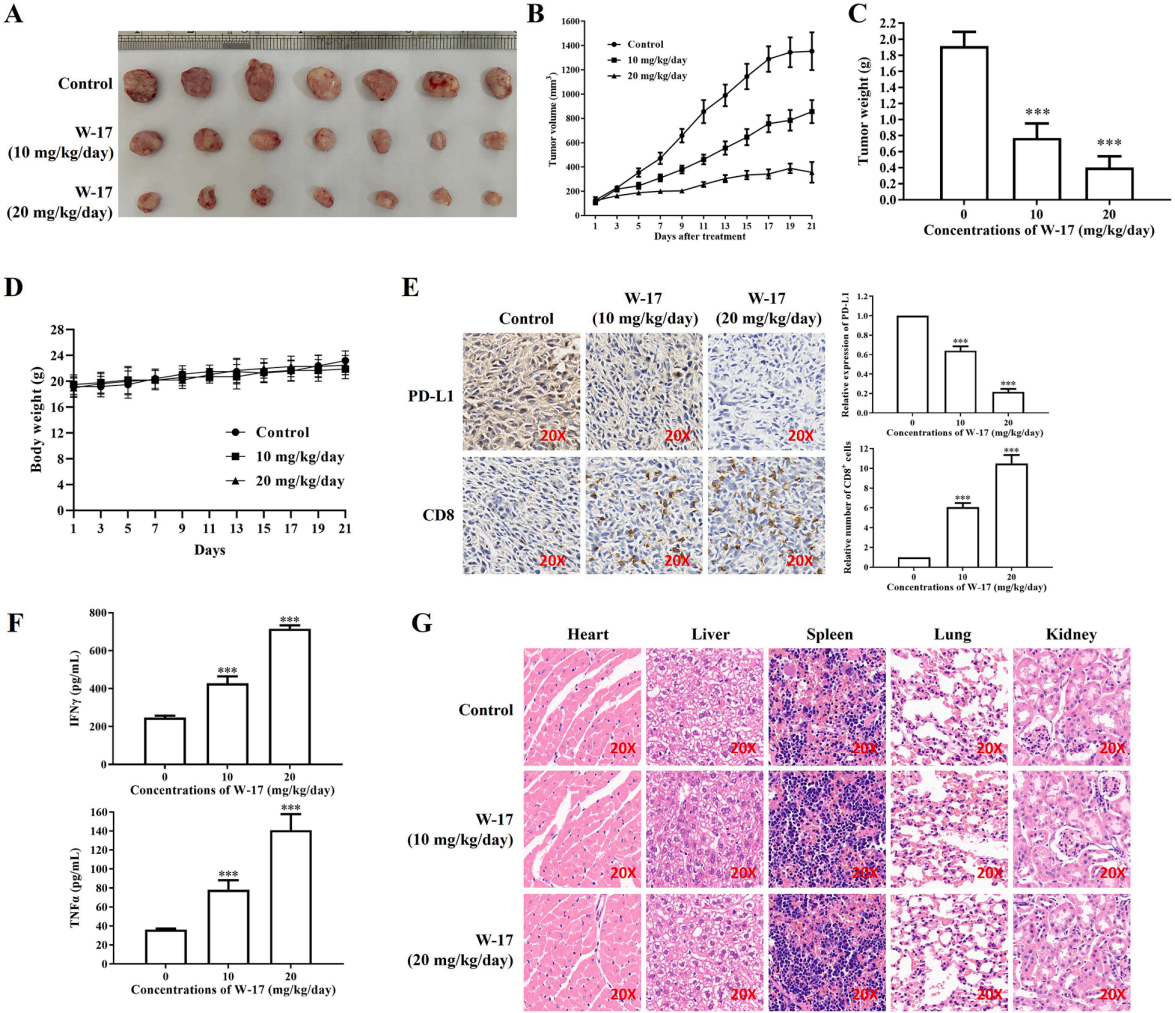
W-17 suppresses tumor growth and promotes immune activation *in vivo*. (A) Representative tumor images from control- and W-17-treated mice at study completion; (B) Longitudinal tumor growth curves showing slowed progression and a dose-dependent response to W-17; (C) Tumor weights are markedly reduced in W-17-treated groups with indicated dosages; (D) Body weight monitoring demonstrating the hardly toxicity of W-17; (E) Immunohistochemical staining of tumor sections revealed decreased PD-L1 expression and increased CD8⁺ T-cell infiltration following W-17 treatment with indicated dosages; (F) ELISA analysis of tumor lysates showing increased IFN-γ and TNF-α levels in W-17-treated mice; (G) H&E staining of heart, liver, spleen, lung, kidney revealed no observable pathological abnormalities. Data were presented as mean ± SD (n = 6); ***p < 0.001 was considered statistically significant.

Building on the functional gains observed in co culture, we tested W-17 in an immunocompetent CT26 colorectal cancer model to determine whether pharmacologic USP8 inhibition translates into tumor control and immune reprogramming in vivo. Mice with established tumors were randomized to vehicle, low dose W-17, or high dose W-17, and dosing was given once daily by intraperitoneal injection for 21 days. Tumor volumes were recorded every two days and body weight was monitored every three days with concurrent clinical observation. At study end the excised tumors showed a clear macroscopic reduction in size in the W-17 groups compared with vehicle as shown in Fig. 6A. Longitudinal tracking confirmed slower growth with W-17 and a dose dependent separation of the curves as shown in Fig. 6B, and terminal tumor weight was reduced in treated cohorts as shown in Fig. 6C while body weight remained within five percent of baseline throughout as shown in Fig. 6D.

Mechanistic biomarkers in the same animals aligned with the proposed pathway because immunohistochemical staining of tumor section showed less PD-L1 protein and higher CD8^+^ cells infiltration. Meanwhile, as shown in Fig. 6F, interferon gamma (IFN-γ) and tumor necrosis factor alpha (TNF-α) were also up-regulated. Multiplex immunofluorescence analysis further demonstrated that PD-L1 signals were predominantly localized in tumor cells with minimal colocalization with CD11c-positive dendritic cells (Figure S4), indicating that the majority of PD-L1 expression detected in this model originated from tumor cells.

In addition to CD8⁺ T cells, we further evaluated other immune cell populations in tumor tissues using multiplex immunofluorescence staining. As shown in Figure S5, CD4⁺ T cells and CD19-positive B cells were detectable in the tumor microenvironment, whereas NK1.1-positive NK-cell infiltration was minimal. No obvious difference in CD4⁺ T-cell infiltration was observed between groups, while B-cell infiltration showed a moderate increase following W-17 treatment. These findings suggest that W-17 may exert broader immunomodulatory effects within the tumor microenvironment, although the primary focus of this study remains USP8-mediated PD-L1 regulation and CD8⁺ T-cell-associated antitumor immunity.

General tolerability was acceptable because blinded histopathology of heart liver spleen lung and kidney showed no treatment related lesions as shown in Fig. 6G. These observations indicate that W-17 monotherapy slows tumor growth, lowers tumor PD-L1, enhances intratumoral CD8⁺ T-cell-associated immune responses, and achieves these effects without overt toxicity under the dosing conditions tested.

## Discussion

Microsatellite-stable colorectal cancer responds poorly to immune checkpoint therapy, and this resistance is increasingly linked to mechanisms that preserve PD-L1 protein stability. In this study, USP8 was found to be elevated in colorectal cancer and associated with immunosuppressive characteristics, suggesting a role in supporting PD-L1 abundance. Using W-17, we demonstrated that USP8 inhibition selectively reduced PD-L1 protein levels without significantly affecting PD-L1 mRNA expression. The increased K48-linked ubiquitination of PD-L1 together with its rescue by proteasome inhibition supports a mechanism in which USP8 stabilizes PD-L1 by preventing its degradative ubiquitination.

The functional consequences of this regulation were evident in the T-cell co-culture system. Tumor cells treated with W-17 became more susceptible to CD8⁺ T-cell-mediated killing and induced increased secretion of effector cytokines, including IFN-γ and TNFα. These effects were markedly attenuated in PD-L1-deficient cells or in cells expressing ubiquitination-resistant PD-L1 mutants, supporting that PD-L1 degradation represents a major mechanism underlying the immunomodulatory activity of W-17.

In addition to PD-L1 regulation, our additional data suggest that USP8 inhibition may also influence broader immune-related signaling pathways. W-17 treatment increased the expression of several MHC-I antigen presentation-related molecules, including HLA-A, HLA-B, HLA-C, and PSMB8, accompanied by increased p65 phosphorylation, suggesting activation of NF-κB-associated immune signaling. Furthermore, W-17 promoted immunogenic cell death (ICD)-associated features, including calreticulin exposure and HMGB1 release. These findings indicate that, besides relieving PD-L1-mediated immune suppression, W-17 may further enhance CD8⁺ T-cell-associated antitumor immunity through modulation of IFN/MHC-I-associated antigen presentation pathways and immune-relevant tumor cell death.

The *in vivo* results aligned with these observations. W-17 slowed tumor growth, reduced PD-L1 expression in tumor tissue, and increased infiltration of CD8⁺ T cells together with elevated cytokines, without producing detectable toxicity in major organs. Together, these results identify USP8 as an important regulator of tumor immune evasion and support USP8 inhibition as a promising strategy for enhancing antitumor immunity in microsatellite-stable colorectal cancer.

Several limitations should also be acknowledged. Although our data support that W-17 exerts its antitumor effects at least partially through USP8-dependent PD-L1 regulation, additional in vivo studies using USP8-deficient or rescue tumor models will be necessary to further establish the precise contribution of USP8-dependent immune regulation and tumor cell death pathways. In addition, the potential interplay between USP8 inhibition, ferroptosis, and antitumor immunity warrants further investigation.

Overall, W-17 represents a promising USP8-targeting chemical scaffold that may simultaneously modulate PD-L1 stability, antigen presentation, and immune-relevant tumor cell death, thereby enhancing antitumor immune responses in colorectal cancer.

## Material and methods

### Chemistry

All reagents and solvents were commercially sourced and utilized without additional purification. Reaction progress was monitored by thin-layer chromatography (TLC) on silica gel-coated glass plates (Qingdao Haiyang Chemical Co., G60F-254), with visualization under UV light (254 nm). Purification via column chromatography employed silica gel (Qingdao Haiyang Chemical Co., 200–300 mesh). Melting points were determined using an X-5 micro melting point apparatus. NMR spectra (^1^H and ^13^C) were recorded on a JEOL 400 MHz spectrometer, with tetramethylsilane (TMS) as an internal standard, in either CDCl_3_ or DMSO‑*d*_6_. Chemical shifts (δ) are reported in parts per million (ppm) downfield from TMS, and coupling constants (J) are given in hertz (Hz). HRMS (High-resolution mass spectra) data were were acquired on Thermo Fisher Q Exactive plus Orbitrap spectrometer via electrospray ionization (ESI). The purity of all final compounds was>95%, which are directed by Agilent LC1260 high-performance liquid chromatograph equipped with an Agilent ZORBAX 883975-902 HPLC column (5 μm, 150 mm × 4.6 mm). A gradient elution was applied, transitioning from 90:10 to 0:100 water (A)/methanol (B) over 10 minutes at a flow rate of 1 mL/min, with UV detection at 254 nm.

**Scheme 1:**
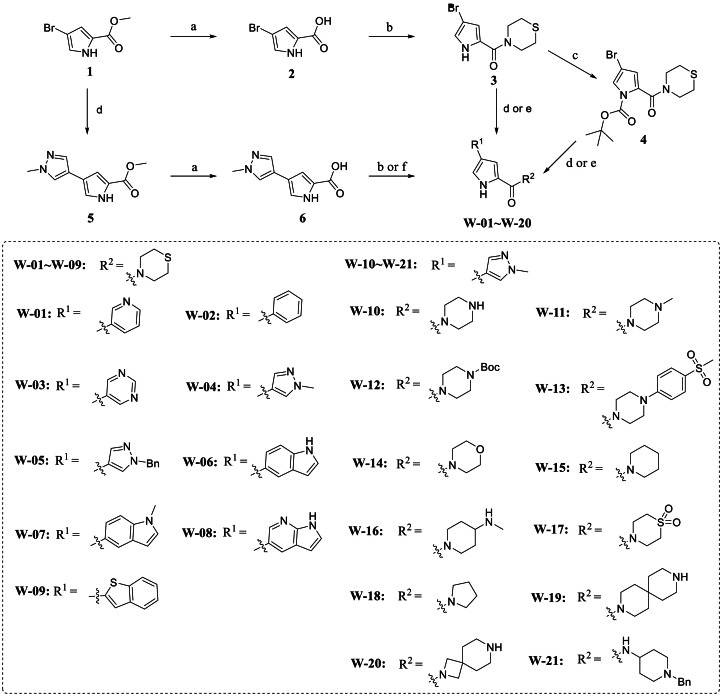
Synthesis route of target compounds W-01∼W-20. Reagents and conditions: (a) NaOH, *V*_EtOH:_*V*_water_ =1:1, 60 °C, 3 h; (b) Secondary amine, HATU, DIPEA, DMF, rt, N_2,_ 6 h; 2) TFA, DCM, rt, 3 h; (c) Di-tert-butyl dicarbonate, TEA, DMAP, THF, rt, 12 h; (d) R^1^B(OH)_2,_ Pd(PPh_3_)_4_, Na_2_CO_3_, DMF, 110 °C, N_2_, 12 h. (e) R^1^BPin, Pd(dppf)Cl_2_, Na_2_CO_3_, *V*_1,4-dioxane_: *V*_water_ =10:1, 100 °C, N_2_, 12 h; (f) Secondary amine, HATU, DIPEA, DMF, rt, N_2,_ 6 h; 2) TFA, DCM, rt, 3 h;.

Target compound W01-21 can be synthesized via the following two routes (Scheme 1): 1. Using the commercially available compound 1 as the starting material, intermediate 2 was prepared by treatment with sodium hydroxide. Intermediate 2 underwent an amide condensation reaction with thiomorpholine to generate intermediate 3. Intermediate 3 was further reacted with di-tert-butyl dicarbonate to yield intermediate 4. Finally, the target compound W01-21 can be prepared by reacting either intermediate 3 or intermediate 4 with boronic acid compounds. 2. Using the commercially available compound 1 as the starting material, intermediate 5 was synthesized by reaction with (1-methyl-1H-pyrazol-4-yl)boronic acid. Intermediate 5 was converted into intermediate 6 after treatment with sodium hydroxide. Intermediate 6 was subsequently reacted with a series of amino compounds, and the target compound W01-21 was obtained eventually.

### Molecular docking

Docking simulations were carried out using MOE 2019 to explore the interaction between W-17 and USP8. The crystal structure of USP8 (PDB entry 3N3K) [28] was employed as the receptor model. Prior to docking, the protein was prepared with the QuickPrep module, during which missing atoms were supplemented, crystallographic water molecules were omitted, and energy minimization was conducted. W-17 was protonated, energetically minimized, and subjected to conformational search to yield a series of low-energy conformers. These conformers were subsequently docked into the predefined ligand-binding pocket of USP8 using the default DOCK protocol. A total of 30 binding poses were generated and ranked according to London dG and GBVI/WSA dG scoring functions. Following visual inspection, the top-ranked pose was selected as the representative binding mode, illuminating the key interactions underlying W-17′s inhibitory activity.

### MD simulations

To assess the dynamic stability of the USP8-W-17 complex, all-atom molecular dynamics (MD) simulations were conducted for 100 ns using the GROMACS 2022 package. Protein topology parameters were derived using the pdb2gmx utility, while ligand parameters were generated via the Antechamber module based on the GAFF2 force field, with partial atomic charges derived from the RESP fitting procedure. The AMBER14SB force field was applied to describe the protein. The system was solvated in a cubic box of TIP3P water molecules with a minimum 1.0 nm distance from the solute to the box edge. Charge neutrality was achieved by adding Na^+^ and Cl^-^ ions to reach a physiological concentration of 0.15 M [30] Long-range electrostatic forces were handled with the particle mesh Ewald (PME) algorithm using a 1.0 nm real-space cutoff, and covalent bonds involving hydrogen atoms were constrained via the LINCS algorithm. Energy minimization was performed sequentially: first 3000 steps of steepest descent, followed by 2000 steps of conjugate gradient, with gradually decreasing positional restraints on the solute. Production MD simulations were conducted under the NPT ensemble at 310 K using the Nosé-Hoover thermostat and 1 bar pressure controlled by the Parrinello-Rahman barostat, with a time step of 2 fs and a total simulation time of 100 ns. Post-simulation trajectory analysis, including RMSD, RMSF, hydrogen bonds, Rg, and SASA, was performed with built-in GROMACS tools to evaluate complex stability and conformational dynamics.

### Cell culture

HCT116, SW480, LoVo, HT29, and NCM460 human cell lines, as well as the murine colorectal cancer cell line CT26, were all purchased from the Cell Bank of the Chinese Academy of Sciences (Shanghai, China). HCT116, SW480, LoVo, HT29, and NCM460 cell lines were grown in Dulbecco’s Modified Eagle’s Medium (DMEM; 11995, Solarbio, China) supplemented with 10% fetal bovine serum (FBS; RYS-KF22, Royabio, China). CT26 cell line was maintained in RPMI-1640 medium (31800, Solarbio, China) with 10% FBS. Cultures were maintained at 37°C in a humidified atmosphere containing 5% CO₂.All these cell lines were yearly verified by short tandem repeat (STR) profiling (Immocell, China) to confirm cell identity throughout the study.

### Prokaryotic expression and purification of USP8_CD_ recombinant

DNA encoding the catalytic turncation of human USP8 was inserted into the pET-28a(+) vector (Novagen, Germany), so that to generate an N-terminal 6 × His-tagged construct. The plasmid was transformed into *E. coli* BL21 (DE3) cells (Novagen, Germany), and cells were then cultured in LB medium supplemented with 50 μg/mL kanamycin (K2011, Saint-Bio, China). Bacterial cultures were grown at 37°C with shaking until reaching an OD_600_ of 0.5, and protein expression was then induced with 0.1 mM IPTG (I8070, Solarbio, China) at 20°C for 12 hours.

Cells were then collected and resuspended in lysis buffer containing 50 mM Tris-HCl (pH 8.0), 150 mM NaCl. Then, cells was sonicated on ice, and the lysates were centrifuged at 15,000 × g for 30 minutes at 4°C. The supernatant was collected, filtered using the 0.2 μm filter, and applied to Ni-NTA resin (SA004005, Smart-lifesciences, China) equilibrated with the same lysis buffer. After washing with buffer containing 25 mM imidazole, the His-tagged USP8_CD_ protein was eluted using 250 mM imidazole.

The collected fraction was then applied to a Superdex 200 column (GE Healthcare, USA) equilibrated with size-exclusion buffer (50 mM HEPES, pH 7.5; 150 mM NaCl; 1 mM DTT) using AKTA system (GE Healthcare, USA). The purity of the purified protein was confimed by SDS–polyacrylamide gels, and the final protein was concentrated to 10 mg/mL with 20% glycerol addition, and stored at -80°C until use.

### AMC-Ub based USP8 assay

USP8 enzymatic activity was assessed using a ubiquitin-AMC fluorescence substrate following standard procedures [17,31,32]. Briefly, Recombinant USP8 catalytic domain protein was first diluted in assay buffer containing 50 mM Tris-HCl (pH 7.5), 2 mM DTT, and 0.1 mg/mL BSA. Test compounds were dissolved in DMSO and incubated with the enzyme solution at room temperature for 15 minutes to allow compound-protein interaction. The reaction was initiated by adding ubiquitin-AMC substrate to a final concentration of 500 nM. Fluorescence release generated by AMC cleavage was monitored immediately using a microplate reader, with excitation at 350 nm and emission at 440 nm. Measurements were recorded at 1 minute intervals for 30 minutes, and reaction velocities were calculated from the linear portion of the fluorescence curves. USP8 activity in the presence of compounds was normalized to vehicle controls containing the same DMSO concentration. IC₅₀ values were determined by nonlinear regression using GraphPad Prism, based on at least three independent assays.

### Western blot

Cells were collected and lysed on ice for 30 minutes using RIPA buffer (P0013K, Beyotime, China) supplemented with protease and phosphatase inhibitor cocktails (HY-K0013, MCE, China). The lysates were centrifuged at 10,000 × g for 30 minutes at 4°C, so that to collect the clear supernatants, and then the supernatant was subjected to BCA assay (PC0020, Solarbio, China) for quantification. After mixing with the denaturing buffer (Solarbio, China) and denatured for 5 min, equal amounts of protein (20-40 μg per sample) were resolved on SDS-polyacrylamide gels and transferred onto nitrocellulose membranes (GE, USA). Membranes were blocked with 5% non-fat milk prepared in TBST (Tris-buffered saline containing 0.1% Tween-20) for 1 hour at room temperature, followed by overnight incubation at 4°C with the indicated primary antibodies. After washing with TBST, membranes were incubated for 1 hour at room temperature with HRP-conjugated secondary antibodies (RGAR001 or RGAM001, Proteintech, China). Protein signals were visualized using enhanced chemiluminescence reagents (34577, Thermo Fisher, USA) and imaged with a Tanon chemiluminescence detection system (Tanon, China).

Antibody selection is as follows: anti-human USP8 (67321-1-Ig, Proteintech, China), anti-human PD-L1 (13684, CST, USA), anti-human Ubiquitin (80992-1-RR, Proteintech, China); anti-human p-P65 (82335-1-RR, Proteintech, China); anti-GAPDH (60004-1-Ig, Proteintech, China).

### Immunoprecipitation

Cells were collected and lysed on ice for 30 minutes in IP buffer (I1030, Solarbio, China) with a protease inhibitor cocktail (HY-K0010, MCE, China). The lysates were centrifuged at 12,000 × g for 15 minutes at 4°C, and the supernatants were collected for immunoprecipitation. For each reaction, 500 μg to 1 mg of protein was incubated with the primary antibodies together with Protein A/G agarose beads (36403ES25, Yeasen, China) overnight at 4°C, while normal IgG (HA722127, Huabio, China) was used as the negative control. After incubation, the beads were washed with ice-cold wash buffer composed of 50 mM Tris-HCl (pH 7.5), 150 mM NaCl, 0.1% NP-40, and 1 mM EDTA. Bound proteins were released by boiling the beads in denaturing buffer (Solarbio, China) for 5 minutes at 95°C, and the eluted samples were subjected to Western blot analysis using the corresponding antibodies.

### T-cell killing assay

For T-cell killing assay, tumor cells were pretreated with W-17 to modulate PD-L1 expression before co-culture, allowing evaluation of how USP8 inhibition influences tumor susceptibility to CD8⁺ T-cell-mediated cytotoxicity. HCT116 cells were seeded in 96-well plates and allowed to adhere overnight before being treated with the indicated concentrations of W-17 for 24 hours. Human CD8⁺ T cells were isolated from peripheral blood using a magnetic-bead based kit (130-045-201, Miltenyi Biotec, Germany) from healthy donors according to the ethical guidelines of our hospital (ethical approval number: HNSRMYY2023051923) Purified T cells were activated for 48 hours with anti-CD3/CD28 beads (422604, BioLegend, USA) in the presence of IL-2. Following activation, T cells were added to tumor cell cultures at the specified effector-to-target ratios (E:T), and co-cultures were maintained for 24 hours. Cell viability was quantified using the CCK-8 kit (CK04, Dojindo, Japan). For experiments examining the effect of PD-L1 loss, PD-L1 knockout cells and corresponding control cells were generated by CRISPR/Cas9 editing (Vectored sgRNA, Tsingke, China) and used under the same conditions. Cytokine levels in the co-culture supernatants were measured by ELISA (MultiSciences, China) to evaluate T-cell activation. All assays were performed in triplicate.

### ELISA

For cytokine quantification, culture supernatants or tumor tissue lysates were collected and centrifuged at 12,000 × g for 30 minutes at 4°C. IFN-γ, TNF-α and HMGB1 were quantified using ELISA kits (EK280HS, EK180HS, EK182HS, EK282HS, MultiSciences, China; HMGB1, COIBO BIO, China). Standards and samples were added to pre-coated 96-well plates, incubated for the recommended times, washed, and treated with HRP-linked detection antibodies. After adding the TMB substrate, reactions were stopped with the provided stop solution, and absorbance was read at 450 nm. Cytokine concentrations were calculated according to the standard curves. All measurements were performed in triplicate.

### Immunohistochemical staining

Tumor tissues were fixed in 4% paraformaldehyde, embedded in paraffin, and sectioned at a thickness of 4 μm. After deparaffinization in xylene and rehydration through graded ethanol, antigen retrieval was performed by heating the sections in citrate buffer (pH 6.0) for 15 minutes. Endogenous peroxidase activity was blocked with 3% hydrogen peroxide, followed by incubation with 5% BSA to reduce nonspecific binding. Sections were then incubated overnight at 4°C with primary antibodies against PD-L1 or CD8 (80517, 98941, CST, USA). After washing, slides were treated with HRP-conjugated secondary antibodies (Proteintech, China) and developed using DAB substrate (ZLI-9018, ZSGB-Bio, China). Hematoxylin was used for counterstaining, and the slides were dehydrated, mounted, and imaged using a light microscope. Positive staining areas were quantified with ImageJ software, and at least three independent fields were analyzed for each sample.

### Flow cytometry

For flow cytometric analysis, treated cells were collected, washed with PBS, and resuspended in staining buffer containing 2% FBS. To assess PD-L1 surface expression, cells were incubated for 30 minutes at 4°C with fluorophore-conjugated anti-PD-L1 antibody (329708, BioLegend, USA), anti-CRT antibody (10292-1-AP, proteintech, China) or the corresponding isotype control. After washing, samples were immediately analyzed using a BD FACSCanto II cytometer. Gating strategies were established based on unstained, single-stained, and isotype controls, and at least 10,000 events were collected for each sample.

### RT-qPCR

Total RNA was isolated from treated cells using RNA extraction kit (RC350-01, Vazyme, China), and the quality control of RNA were performed with a NanoDrop spectrophotometer. 1 μg RNA was reverse-transcribed into cDNA using the HiScript II reverse transcription kit (R201-01, Vazyme, China). Quantitative PCR was performed on a Abi7500 real-time PCR system (Thermo Fisher Scientific, USA) with SYBR Green Master Mix (P111-03, Vazyme, China). Relative mRNA levels were calculated using the 2^⁻ΔΔCt^ method according to standard analytical procedures.

Primers used are as follows:

**Table:** 

Name	Primer-F	Primer-R
PD-L1	5-TGGCATTTGCTGAACGCATTT-3	5-TGCAGCCAGGTCTAATTGTTTT-3
HLA-A	5-ACCCTCGTCCTGCTACTCTC-3	5-CTGTCTCCTCGTCCCAATACT-3
HLA-B	5-CAGTTCGTGAGGTTCGACAG-3	5-CAGCCGTACATGCTCTGGA-3
HLA-C	5-GGACAAGAGCAGAGATACACG-3	5-CAAGGACAGCTAGGACAACC-3
TAP1	5-CTGGGGAAGTCACCCTACC-3	5-CAGAGGCTCCCGAGTTTGTG-3
TAP2	5-TGGACGCGGCTTTACTGTG-3	5-GCAGCCCTCTTAGCTTTAGCA-3
PSMB8	5-TCTCCAGAGCTCGCTTTACC-3	5-CACTCCATGCTGGAACTTGA-3
PSMB9	5-CGTTGTGATGGGTTCTGATTCC-3	5-GACAGCTTGTCAAACACTCGGTT-3
GAPDH	5-GGAGCGAGATCCCTCCAAAAT-3	5-GGCTGTTGTCATACTTCTCATGG-3

### In vivo xenograft model

Female BALB/c mice aged 6-8 weeks were obtained from SPF Biotechnology (Beijing, China) and housed under specific pathogen-free conditions with unrestricted access to food and water. CT26 cells were suspended in PBS, and 5 × 10⁵ cells were injected subcutaneously into the right flank to establish tumors. When tumor volumes reached approximately 80-100 mm³, animals were randomly allocated to treatment groups and administered W-17 at the indicated doses by intraperitoneal injection every two days; control mice received vehicle following the same schedule. Tumor dimensions were recorded with calipers at two-day intervals, and volumes were calculated using the formula (length × width²)/2. In the end of the study, mice were euthanized, and tumors were stripped and weighed; organs were collected for histopathological evaluation. All procedures conformed to institutional animal care guidelines and were approved by the Animal Ethics Committee of Zhengzhou University under approval number ZZU-2023-102.

### Statistical analysis

Statistical analyses were carried out using GraphPad Prism 9.0. Data are presented as mean ± SD unless otherwise stated. Comparisons between two groups were performed using unpaired two-tailed Student’s *t* tests, while multiple-group comparisons were evaluated by one-way ANOVA followed by appropriate post hoc testing. Tumor growth curves were analyzed by two-way ANOVA with repeated measures. All experiments were independently repeated at least three times, and P values < 0.05 were considered statistically significant.

## CRediT authorship contribution statement

**Zhiyu Song:** Writing – original draft, Methodology, Formal analysis, Data curation. **Yuanying Wang:** Methodology, Investigation, Formal analysis, Data curation, Conceptualization. **Weiwei Zhou:** Validation, Software, Resources. **Xiaomei Huang:** Writing – original draft, Visualization, Validation. **Gang Jia:** Writing – original draft, Visualization, Conceptualization. **Mingyang Han:** Writing – review & editing, Writing – original draft, Funding acquisition, Conceptualization.

## Declaration of competing interest

The authors declare that they have no known competing financial interests or personal relationships that could have appeared to influence the work reported in this paper.

## Appendix Supplementary materials

Image, application 1
