# Supplementary material for: Discovery of indazole-carboxamide based USP8 inhibitor for immune activation and antitumor response in colorectal cancer
Source: Transl Oncol. 2026 Aug 5;72:102956. doi: 10.1016/j.tranon.2026.102956 (PMC13471239; doi:10.1016/j.tranon.2026.102956)
Supplement: Supplementary file 1 [file mmc1.docx]

**Supplementary Information**

**Discovery of Indazole-Carboxamide Based USP8 Inhibitor for Immune Activation and Antitumor Response in Colorectal Cancer**

Zhiyu Song^1, #^, Yuanying Wang^2, #^, Weiwei Zhou^2^, Xiaomei Huang^2^, Gang Jia^3^, Mingyang Han^4,^ *

1 Department of Pharmacy, Henan Provincial People's Hospital; People's Hospital of Zhengzhou University, Zhengzhou, Henan, 450003, China.

2 People's Hospital of Zhengzhou University, Zhengzhou, Henan, 450003, China.

3 Department of oncology, Henan Provincial People's Hospital;People's Hospital of Zhengzhou University, Zhengzhou, Henan, 450003, China.

4 Department of Gastrointestinal Surgery, Henan Provincial People's Hospital; People's Hospital of Zhengzhou University, Zhengzhou, Henan, 450003, China

^#^ These authors contributed equally.

^*^ Corresponding author: Zhiyu Song, songzy@zzu.edu.cn

**Table S1 and Figure S1-5……………………………………………...……..…….S2**

**Synthetic procedure and characterization of target compounds..........................S5**

**^1^H NMR and ^13^C NMR spectra of compounds……………………………..……S14**

**HRMS spectra of compounds…………………………………………………….S43**

**HPLC spectra of target compounds………………………...............................…S49**

**Original immunoblot data………..……………………….....................................S60**

Table S1. Inhibitory activities of W-17 against USP7, USP10 and USP14.

| Proteins | IC_50_ (μM) |
| --- | --- |
| USP7 | ＞10 |
| USP10 | ＞10 |
| USP14 | ＞10 |

**Figure S1** USP8 deficiency attenuates W-17-mediated CD8⁺ T-cell-associated antitumor immune responses. (A) Flow cytometry analysis of PD-L1 expression in USP8-knockout colorectal cancer cells HCT-116 treated with the indicated concentrations; (B) Survival ratio of USP8-knockout colorectal cancer cells after co-culture with activated CD8⁺ T cells at different effector-to-target (E:T) ratios; (C) ELISA analysis of IFN-γ and TNF-α levels in the co-culture supernatants. Data were presented as mean ± SD; * p < 0.05 was considered statistically significant.

**Figure S2** W-17 induces ICD-associated features in gastric cancer cells. (A) Flow cytometry analysis of calreticulin (CRT) exposure on the surface of gastric cancer cells following treatment with the indicated concentrations of W-17 for 24 h. Quantification of CRT-positive cells is shown in each panel. (B) ELISA analysis of HMGB1 release in the culture supernatants of gastric cancer cells treated with the indicated concentrations of W-17 for 24 h. Data were presented as mean ± SD; ** p < 0.01, *** p < 0.001 was considered statistically significant.

**Figure S3** W-17 enhances MHC-I-associated antigen presentation signaling in gastric cancer cells. (A) qPCR analysis of MHC-I antigen presentation-related genes in gastric cancer cells treated with W-17 (4 μM). Relative mRNA expression levels of HLA-A, HLA-B, HLA-C, TAP1, TAP2, PSMB8, and PSMB9 were normalized to the control group; (B) Western blot analysis of phosphorylated p65 (p-p65) in gastric cancer cells treated with the indicated concentrations of W-17. GAPDH was used as the loading control. Data were presented as mean ± SD; *** p < 0.001 was considered statistically significant.

**Figure S4** Multiplex immunofluorescence staining in tumor tissues from the control group. PD-L1 (green), CD11c-positive dendritic cells (cyan), and nuclei stained with DAPI (blue) are shown. Representative images indicate that PD-L1 signal was predominantly localized in tumor cells and showed minimal colocalization with CD11c-positive dendritic cells.

**Figure S5** Multiplex immunofluorescence staining in tumor tissues. CD4 (green), CD19-positive B cells (orange), NK1.1-positive NK cells (red) and nuclei stained with DAPI (blue) are shown.

**Synthetic procedure and characterization of target compounds**

Stir the mixture of Reactant 1 (15.0 g, 73.52 mmol), NaOH (5.88 g, 147.04 mmol), ethanol (150 mL) and water (150 mL) at 60 °C for 3 hours. Cool the mixture to room temperature, and adjust the pH of the reaction solution to 3–4 with 1 N HCl. Filter the precipitated solid by suction, wash with water and dry to obtain the target compound 2.

4-Bromo-1H-pyrrole-2-carboxylic acid (2). white solid; mp 184.9-185.7 ℃; yield, 71.6%; ^1^H NMR (400 MHz, DMSO-*d*_6_) δ 12.06 (s, 1H), 7.06 (dd, *J* = 3.1, 1.7 Hz, 1H), 6.71-6.67 (m, 1H). ^13^C NMR (100 MHz, DMSO-*d*_6_) δ 161.51, 124.34, 123.76, 116.21, 96.15.

Dissolve reactant 2 (10.00 g, 52.63 mmol) in DMF (200 mL). Add HATU (30.02 g, 78.95 mmol) and DIPEA (20.41 g, 157.90 mmol), and stir the mixture at room temperature for 20 min under a nitrogen atmosphere. Subsequently, add thiomorpholine (8.15 g, 78.95 mmol) and continue stirring at room temperature for 6 hours under a nitrogen atmosphere. Upon completion of the reaction, extract the mixture with water and ethyl acetate (100 mL × 3). Dry the organic phase over anhydrous magnesium sulfate, concentrate the solution, and purify the residue by column chromatography (petroleum ether: ethyl acetate = 5:1) to afford compound 3.

(4-Bromo-1H-pyrrol-2-yl)(thiomorpholino)methanon (3). yellow solid; mp 178.7-179.6 ℃; yield, 27.6%; ^1^H NMR (400 MHz, DMSO-*d*_6_) δ 11.78 (s, 1H), 6.99 (dd, *J* = 3.1, 1.5 Hz, 1H), 6.50 (dd, *J* = 2.7, 1.5 Hz, 1H), 3.88-3.81 (m, 4H), 2.63-2.59 (m, 4H). ^13^C NMR (100 MHz, DMSO-*d*_6_) δ 161.09, 125.32, 121.66, 113.42, 95.42, 27.47.

Dissolve reactant 3 (4.00 g, 14.54 mmol) in THF (80 mL). Add di-tert-butyl dicarbonate (3.49 g, 15.99 mmol), TEA (1.77 g, 17.44 mmol) and DMAP (355.19 mg, 2.91 mmol), then stir the mixture at room temperature for 12 hours. Upon completion of the reaction, add saturated aqueous sodium bicarbonate solution (50 mL), followed by extraction with ethyl acetate (50 mL × 3). Dry the organic layer over anhydrous magnesium sulfate, filter, concentrate the filtrate, and purify the residue by column chromatography (petroleum ether: ethyl acetate = 15:1) to afford compound 4.

Tert-butyl 4-bromo-2-(thiomorpholine-4-carbonyl)-1H-pyrrole-1-carboxylate (4). white solid; mp 95.3-96.5 ℃; yield, 73.3%; ^1^H NMR (400 MHz, DMSO-*d*_6_) δ 7.42 (d, *J* = 1.5 Hz, 1H), 6.49 (d, *J* = 1.7 Hz, 1H), 3.76 (s, 2H), 3.50 (s, 2H), 2.60 (s, 2H), 2.54 (s, 2H), 1.45 (s, 9H). ^13^C NMR (100 MHz, DMSO-*d*_6_) δ 161.32, 147.16, 128.65, 121.43, 115.04, 99.52, 85.95, 49.73, 44.27, 27.75, 27.24, 26.86.

Dissolve reactant 1 (10.00 g, 49.01 mmol) in DMF (250 mL). Add (1-methyl-1H-pyrazol-4-yl)boronic acid (15.43 g, 122.54 mmol), 2 N Na_2_CO_3_ (20.78 g, 196.06 mmol) and Pd(PPh_3_)_4_ (5.66 g, 4.90 mmol). Stir the mixture at 110 °C for 8 hours under a nitrogen atmosphere. Upon completion of the reaction, extract the mixture with water and ethyl acetate (150 mL × 3). Dry the organic layer over anhydrous magnesium sulfate, filter, concentrate the filtrate, and purify the residue by column chromatography (petroleum ether: ethyl acetate = 5:1) to afford compound 5.

Methyl 4-(1-methyl-1H-pyrazol-4-yl)-1H-pyrrole-2-carboxylate (5). white solid; mp 176.9-177.7 ℃; yield, 69.6%; ^1^H NMR (400 MHz, DMSO-*d*_6_) δ 11.82 (s, 1H), 7.80 (s, 1H), 7.59 (d, *J* = 0.9 Hz, 1H), 7.17 (d, *J* = 1.7 Hz, 1H), 6.90 (d, *J* = 1.7 Hz, 1H), 3.76 (s, 3H), 3.72 (s, 3H). ^13^C NMR (100 MHz, DMSO-*d*_6_) δ 161.28, 136.34, 127.15, 122.44, 120.73, 117.77, 116.84, 112.72, 51.56, 38.96.

Stir the mixture of Reactant 5 (7.00 g, 34.11 mmol), NaOH (2.73 g, 68.22 mmol), ethanol (70 mL) and water (70 mL) at 60 °C for 3 hours. Cool the mixture to room temperature, and adjust the pH of the reaction solution to 3-4 with 1 N HCl. Filter the precipitated solid by suction, wash with water and dry to afford 6.

4-(1-Methyl-1H-pyrazol-4-yl)-1H-pyrrole-2-carboxylic acid (6). white solid; mp 205.9-207.1 ℃; yield, 61.3%; ^1^H NMR (400 MHz, DMSO-*d*_6_) δ 11.61 (s, 1H), 7.78 (d, *J* = 5.8 Hz, 1H), 7.56 (d, *J* = 5.7 Hz, 1H), 7.10 (s, 1H), 6.81 (s, 1H), 3.75 (s, 3H). ^13^C NMR (100 MHz, DMSO-*d*_6_) δ 162.34, 136.27, 127.11, 123.58, 120.08, 117.47, 117.03, 112.40, 38.94.

Dissolve reactant 4 (200.00 mg, 532.93 μmol) in DMF (5 mL). Add R^1^B(OH)_2_ (1.33 mmol), Na_2_CO_3_ (508.36 mg, 4.80 mmol) and Pd(PPh_3_)_4_ (30.79 mg, 26.65 μmol). Under a nitrogen atmosphere, stir the reaction mixture at 110 °C for 10 hours. After the reaction is complete, extract the mixture with water and ethyl acetate (20 mL × 3 times). Dry the organic phase over anhydrous magnesium sulfate, filter the mixture, and concentrate the filtrate under reduced pressure. Purify the residue by column chromatography (petroleum ether: ethyl acetate = 1:1) to obtain the target compounds W-01~04, W-06~07, W-09..

(4-(Pyridin-3-yl)-1H-pyrrol-2-yl)(thiomorpholino)methanone (W-01). white solid; mp 243.9-245.1 ℃; yield, 27.5%; ^1^H NMR (400 MHz, DMSO-*d*_6_) δ 11.74 (s, 1H), 8.85 (d, *J* = 2.4 Hz, 1H), 8.31 (dd, *J* = 4.7, 1.7 Hz, 1H), 7.95 (dt, *J* = 7.9, 2.0 Hz, 1H), 7.47 (dd, *J* = 3.1, 1.5 Hz, 1H), 7.29 (dd, *J* = 7.9, 4.7 Hz, 1H), 6.95-6.91 (m, 1H), 3.92 (s, 4H), 2.68-2.63 (m, 4H). ^13^C NMR (100 MHz, DMSO-*d*_6_) δ 161.88, 146.97, 146.66, 132.22, 131.33, 125.99, 124.15, 121.17, 119.62, 109.43, 27.56. HR-MS (ESI): Calcd. C_14_H_15_N_3_O_S_ [M + H]^+^ m/z: 274.10086, found: 274.10081. HPLC retention time = 4.638 min, purity = 98.15% (λ = 254 nm).

(4-Phenyl-1H-pyrrol-2-yl)(thiomorpholino)methanone (W-02). white solid; mp 138.4-139.7 ℃; yield, 11.5%; ^1^H NMR (400 MHz, DMSO-*d*_6_) δ 11.60 (s, 1H), 7.56 (d, *J* = 7.5 Hz, 2H), 7.34 (d, *J* = 7.5 Hz, 1H), 7.27 (t, *J* = 7.7 Hz, 2H), 7.10 (t, *J* = 7.4 Hz, 1H), 6.83 (d, *J* = 9.9 Hz, 1H), 3.93 (s, 4H), 2.65 (s, 4H). ^13^C NMR (100 MHz, DMSO-*d*_6_) δ 162.03, 135.58, 129.11, 125.97, 125.60, 125.26, 124.57, 118.94, 109.37, 27.55. HR-MS (ESI): Calcd. C_15_H_16_N_2_OS [M + H]^+^ m/z: 273.10561, found: 273.10561. HPLC retention time = 4.351 min, purity = 96.48% (λ = 254 nm).

(4-(Pyrimidin-5-yl)-1H-pyrrol-2-yl)(thiomorpholino)methanone (W-03). white solid; mp 254.4-256.2 ℃; yield, 23.9%; ^1^H NMR (400 MHz, DMSO-*d*_6_) δ 11.87 (s, 1H), 9.06 (s, 2H), 8.91 (s, 1H), 7.60 (dd, *J* = 3.1, 1.6 Hz, 1H), 7.05-7.02 (m, 1H), 3.92 (s, 4H), 2.69-2.63 (m, 4H). ^13^C NMR (100 MHz, DMSO-*d*_6_) δ 161.71, 155.94, 153.14, 129.47, 126.35, 120.31, 117.60, 109.41, 27.56. Calcd. C_13_H_14_N_4_OS [M + H]^+^ m/z: 275.09611, found: 275.09599. HPLC retention time = 4.149 min, purity = 99.01% (λ = 254 nm).

(4-(1-Methyl-1H-pyrazol-4-yl)-1H-pyrrol-2-yl)(thiomorpholino)methanone (W-04). white solid; mp 226.7-228.1 ℃; yield, 18.7%; ^1^H NMR (400 MHz, DMSO-*d*_6_) δ 11.36 (s, 1H), 7.77 (s, 1H), 7.56 (s, 1H), 7.03 (dd, *J* = 2.9, 1.5 Hz, 1H), 6.57 (dd, *J* = 2.6, 1.7 Hz, 1H), 3.93-3.87 (m, 4H), 3.76 (s, 3H), 2.66-2.61 (m, 4H). ^13^C NMR (100 MHz, DMSO-*d*_6_) δ 162.00, 136.30, 127.04, 124.84, 117.90, 117.25, 116.64, 109.71, 38.94, 27.52. HR-MS (ESI): Calcd. C_13_H_16_N_4_OS, [M + H]^+^ m/z: 277.11176, found: 277.11204. HPLC retention time = 4.193 min, purity = 99.54% (λ = 254 nm).

(4-(1H-indol-5-yl)-1H-pyrrol-2-yl)(thiomorpholino)methanone (W-06). brown solid; mp 224.7-225.6 ℃; yield, 24.1%; ^1^H NMR (400 MHz, DMSO-*d*_6_) δ 11.44 (s, 1H), 10.94 (s, 1H), 7.71 (d, *J* = 6.1 Hz, 1H), 7.31 (d, *J* = 6.0 Hz, 2H), 7.27-7.20 (m, 2H), 6.80 (d, *J* = 8.7 Hz, 1H), 6.35 (d, *J* = 8.9 Hz, 1H), 4.01-3.90 (m, 4H), 2.74-2.62 (m, 4H). ^13^C NMR (100 MHz, DMSO-*d*_6_) δ 162.15, 135.11, 128.59, 126.59, 126.29, 126.01, 125.16, 119.78, 117.91, 116.34, 111.95, 109.39, 101.51, 27.58. HR-MS (ESI): Calcd. C_17_H_17_N_3_OS, [M + H]^+^ m/z: 312.11651, found: 312.11631. HPLC retention time = 3.996 min, purity = 98.13% (λ = 254 nm).

(4-(1-Methyl-1H-indol-5-yl)-1H-pyrrol-2-yl)(thiomorpholino)methanone (W-07). gray solid; mp 221.5-222.7 ℃; yield, 6.34%; ^1^H NMR (400 MHz, DMSO-*d*_6_) δ 11.43 (s, 1H), 7.71 (d, *J* = 1.7 Hz, 1H), 7.41-7.30 (m, 2H), 7.26-7.20 (m, 2H), 6.80 (t, *J* = 2.5 Hz, 1H), 6.33 (d, *J* = 3.1 Hz, 1H), 3.94 (s, 4H), 3.73 (s, 3H), 2.69-2.63 (m, 4H). ^13^C NMR (100 MHz, DMSO-*d*_6_) δ 162.14, 135.68, 130.32, 128.95, 126.71, 126.08, 125.21, 119.78, 118.02, 116.58, 110.24, 109.39, 100.72, 33.01, 27.56. HR-MS (ESI): Calcd. C_18_H_19_N_3_OS, [M + H]^+^ m/z: 326.13216, found: 326.13220. HPLC retention time = 4.157 min, purity = 97.39% (λ = 254 nm).

(4-(Benzo[b]thiophen-2-yl)-1H-pyrrol-2-yl)(thiomorpholino)methanone (W-09). gray solid; mp 269.4-272.6 ℃; yield, 33.5%; ^1^H NMR (400 MHz, DMSO-*d*_6_) δ 11.79 (s, 1H), 7.83 (d, *J* = 7.9 Hz, 1H), 7.68 (d, *J* = 7.8 Hz, 1H), 7.47 (s, 1H), 7.36-7.26 (m, 2H), 7.21 (t, *J* = 7.6 Hz, 1H), 6.81 (s, 1H), 4.00-3.83 (m, 4H), 2.72-2.58 (m, 4H). ^13^C NMR (100 MHz, DMSO-*d*_6_) δ 161.69, 141.13, 139.03, 138.04, 126.05, 125.06, 124.07, 123.16, 122.71, 119.82, 118.71, 117.69, 109.82, 27.53. HR-MS (ESI): Calcd. C_17_H_16_N_2_OS_2_, [M + H]^+^ m/z: 329.07768, found: 329.07772. HPLC retention time = 4.170 min, purity = 100.00% (λ = 254 nm).

Dissolve Reactant 4 (200.00 mg, 532.93 μmol) in a mixed solvent of 1,4-dioxane/water (10 mL:1 mL). Add R^1^BPin (799.40 μmol), Na₂CO₃ (169.45 mg, 1.60 mmol) and Pd(dppf)Cl₂ (19.50 mg, 26.65 μmol). Stir the mixture at 100 °C for 10 hours under a nitrogen atmosphere. Upon completion of the reaction, extract the mixture with water and ethyl acetate (20 mL × 3). Dry the organic layer over anhydrous magnesium sulfate, filter, concentrate the filtrate, and purify the residue by column chromatography (petroleum ether: ethyl acetate = 1:1) to afford the target compounds W-05, 08.

(4-(1-Benzyl-1H-pyrazol-4-yl)-1H-pyrrol-2-yl)(thiomorpholino)methanone (W-05). white solid; mp 229.8-230.9 ℃; yield, 15.9%; ^1^H NMR (400 MHz, DMSO-*d*_6_) δ 11.37 (s, 1H), 7.91 (d, *J* = 0.8 Hz, 1H), 7.63 (d, *J* = 0.9 Hz, 1H), 7.33-7.27 (m, 2H), 7.27-7.22 (m, 1H), 7.21-7.17 (m, 2H), 7.05 (dd, *J* = 3.0, 1.5 Hz, 1H), 6.60 (dd, *J* = 2.8, 1.5 Hz, 1H), 5.25 (s, 2H), 3.89 (s, 4H), 2.62 (t, *J* = 5.2 Hz, 4H). ^13^C NMR (100 MHz, DMSO-*d*_6_) δ 162.03, 135.58, 129.11, 125.97, 125.60, 125.26, 124.57, 118.94, 109.37, 27.55. HR-MS (ESI): Calcd. C_19_H_20_N_4_OS, [M + H]^+^ m/z: 353.14306, found: 353.14282. HPLC retention time = 4.086 min, purity = 95.01% (λ = 254 nm).

(4-(1H-pyrrolo[2,3-b]pyridin-5-yl)-1H-pyrrol-2-yl)(thiomorpholino)methanone (W-08). white solid; mp 297.6-298.8 ℃; yield, 8.81%; ^1^H NMR (400 MHz, DMSO-*d*_6_) δ 11.55 (s, 1H), 11.48 (s, 1H), 8.47 (d, *J* = 2.1 Hz, 1H), 8.09 (d, *J* = 2.3 Hz, 1H), 7.37 (d, *J* = 2.6 Hz, 1H), 7.34 (d, *J* = 1.5 Hz, 1H), 6.87 (s, 1H), 6.38-6.35 (m, 1H), 3.95 (s, 4H), 2.67 (t, *J* = 5.8 Hz, 4H). ^13^C NMR (100 MHz, DMSO-*d*_6_) δ 162.03, 147.76, 140.97, 126.85, 125.48, 124.32, 123.63, 123.12, 120.13, 118.33, 109.46, 100.15, 27.57. HR-MS (ESI): Calcd. C_16_H_16_N_4_OS, [M + H]^+^ m/z: 313.11176, found: 313.11169. HPLC retention time = 4.129 min, purity = 99.05% (λ = 254 nm).

Dissolve Reactant 6 (200.00 mg, 1.05 mmol) in DMF (5 mL). Add HATU (596.63 mg, 1.57 mmol) and DIPEA (405.61 mg, 3.14 mmol), and stir the mixture at room temperature for 20 min under a nitrogen atmosphere. Subsequently, add Secondary amine (1.57 mmol) and continue stirring at room temperature for 6 hours under a nitrogen atmosphere. Upon completion of the reaction, extract the mixture with water and ethyl acetate (25 mL × 3). Dry the organic phase over anhydrous magnesium sulfate, concentrate the filtrate, and purify the residue by column chromatography (dichloromethane:methanol = 15:1) to afford compound W-12 (W-10-boc), W-16-boc, W-19-boc, W-20-boc. Dissolve the intermediate compound (200.00 mg) in DCM (10 mL), add trifluoroacetic acid (1 mL), and stir the mixture at room temperature for 3 hours. Upon completion of the reaction, adjust the pH of the mixture to 8 with 1 mol/L sodium hydroxide solution, concentrate the mixture under reduced pressure, and purify the residue by column chromatography (dichloromethane: methanol = 5:1) to afford the target compound W-10, W-16, W-19, W-20.

Tert-butyl 4-(4-(1-methyl-1H-pyrazol-4-yl)-1H-pyrrole-2-carbonyl)piperazine-1-carboxylate (W-12). yellow solid; mp 270.8-272.3 ℃; yield, 44.3%; ^1^H NMR (400 MHz, DMSO-*d*_6_) δ 11.37 (s, 1H), 7.77 (s, 1H), 7.56 (s, 1H), 7.03 (dd, *J* = 2.9, 1.5 Hz, 1H), 6.65 (dd, *J* = 2.8, 1.5 Hz, 1H), 3.76 (s, 3H), 3.65 (s, 4H), 3.37 (s, 4H), 1.38 (s, 9H). ^13^C NMR (100 MHz, DMSO-*d*_6_) δ 161.90, 154.38, 136.29, 127.01, 124.80, 117.95, 117.31, 116.70, 110.16, 79.68, 38.94, 28.58. HR-MS (ESI): Calcd. C_18_H_25_N_5_O_3_, [M + H]^+^ m/z: 360.20301, found: 360.20302. HPLC retention time = 3.998 min, purity = 99.35% (λ = 254 nm).

Tert-butyl methyl(1-(4-(1-methyl-1H-pyrazol-4-yl)-1H-pyrrole-2-carbonyl)piperidin-4-yl)carbamate (W-16-boc). yellow solid; mp 277.4.5-279.3 ℃; yield, 41.1%; ^1^H NMR (400 MHz, DMSO-*d*_6_) δ 11.33 (s, 1H), 7.77 (s, 1H), 7.56 (s, 1H), 7.01 (dd, *J* = 2.9, 1.5 Hz, 1H), 6.60 (dd, *J* = 2.6, 1.5 Hz, 1H), 4.48 (d, *J* = 12.5 Hz, 2H), 4.07 (q, *J* = 5.2 Hz, 1H), 3.76 (s, 3H), 3.13 (d, *J* = 5.2 Hz, 2H), 2.64 (d, *J* = 5.3 Hz, 3H), 1.58 (s, 4H), 1.37 (s, 9H). ^13^C NMR (100 MHz, DMSO-*d*_6_) δ 161.73, 155.06, 136.27, 127.01, 125.07, 117.63, 117.33, 116.53, 109.66, 79.17, 49.13, 38.92, 38.76, 29.01, 28.62. HPLC retention time = 4.129 min, purity = 99.05% (λ = 254 nm).

Tert-butyl 9-(4-(1-methyl-1H-pyrazol-4-yl)-1H-pyrrole-2-carbonyl)-3,9-diazaspiro[5.5]undecane-3-carboxylate (W-19-boc). yellow solid; mp 263.5-265.1 ℃; yield, 44.7%; ^1^H NMR (400 MHz, DMSO-*d*_6_) δ 11.29 (s, 1H), 7.76 (s, 1H), 7.54 (s, 1H), 7.00 (d, *J* = 1.5 Hz, 1H), 6.56 (d, *J* = 1.5 Hz, 1H), 3.76 (s, 3H), 3.63 (s, 3H), 3.28 (s, 4H), 1.44 (t, *J* = 5.7 Hz, 4H), 1.39 (d, *J* = 3.8 Hz, 4H), 1.35 (s, 9H). ^13^C NMR (100 MHz, DMSO-*d*_6_) δ 161.69, 154.50, 136.24, 126.98, 125.30, 117.47, 117.37, 116.45, 109.45, 78.98, 38.92, 38.77, 35.55, 35.28, 30.68, 28.63.

Tert-butyl 2-(4-(1-methyl-1H-pyrazol-4-yl)-1H-pyrrole-2-carbonyl)-2,7-diazaspiro[3.5]nonane-7-carboxylate (W-20-boc). yellow solid; mp 277.5-278.9 ℃; yield, 47.9%; ^1^H NMR (400 MHz, DMSO-*d*_6_) δ 11.41 (s, 1H), 7.77 (s, 1H), 7.56 (s, 1H), 7.03 (dd, *J* = 2.9, 1.5 Hz, 1H), 6.61-6.56 (m, 1H), 4.09 (s, 2H), 3.76 (s, 3H), 3.68 (s, 2H), 3.28-3.21 (m, 5H), 1.63 (t, *J* = 5.9 Hz, 4H), 1.36 (s, 9H). ^13^C NMR (100 MHz, DMSO-*d*_6_) δ 163.10, 161.93, 154.52, 136.24, 126.98, 124.92, 118.18, 117.32, 109.71, 79.21, 38.94, 34.52, 28.60.

(4-(1-Methyl-1H-pyrazol-4-yl)-1H-pyrrol-2-yl)(piperazin-1-yl)methanone (W-10). yellow oily substance; yield, 77.8%. ^1^H NMR (400 MHz, DMSO-*d*_6_) δ 11.45 (s, 1H), 7.78 (s, 1H), 7.56 (s, 1H), 7.10-7.05 (m, 1H), 6.75-6.70 (m, 1H), 3.90-3.83 (m, 4H), 3.76 (s, 3H), 3.19-3.11 (m, 4H). ^13^C NMR (100 MHz, DMSO-*d*_6_) δ 162.04, 159.36, 159.05, 158.74, 158.42, 136.28, 127.07, 124.15, 119.19, 118.40, 117.19, 116.84, 116.22, 113.24, 110.53, 43.28, 38.91. HR-MS (ESI): Calcd. C_13_H_17_N_5_O, [M + H]^+^ m/z: 260.15059, found: 260.15069. HPLC retention time = 4.144 min, purity = 96.42% (λ = 254 nm).

(4-(1-Methyl-1H-pyrazol-4-yl)-1H-pyrrol-2-yl)(4-(methylamino)piperidin-1-yl)methanone (W-16). yellow oily substance; yield, 78.6%. ^1^H NMR (400 MHz, DMSO-*d*_6_) δ 11.39 (s, 1H), 7.78 (s, 1H), 7.56 (s, 1H), 7.03 (dd, *J* = 3.0, 1.5 Hz, 1H), 6.64-6.60 (m, 1H), 4.46 (d, *J* = 13.4 Hz, 2H), 3.76 (s, 3H), 3.23 (d, *J* = 11.2 Hz, 1H), 2.95 (s, 2H), 2.54 (s, 3H), 2.05 (d, *J* = 9.0 Hz, 2H), 1.43 (d, *J* = 12.1 Hz, 2H). ^13^C NMR (100 MHz, DMSO-*d*_6_) δ 161.75, 159.31, 159.00, 158.69, 158.38, 136.27, 127.06, 124.75, 122.22, 119.24, 117.94, 117.25, 116.66, 116.26, 113.28, 109.87, 55.55, 38.92, 29.86, 28.59. HR-MS (ESI): Calcd. C_15_H_21_N_5_O, [M + H]^+^ m/z: 288.18189, found: 288.18192. HPLC retention time = 4.451 min, purity = 96.10% (λ = 254 nm).

(4-(1-Methyl-1H-pyrazol-4-yl)-1H-pyrrol-2-yl)(3,9-diazaspiro[5.5]undecan-3-yl)methanone (W-19). yellow solid; mp 166.2-167.8 ℃; yield, 56.9%; ^1^H NMR (400 MHz, DMSO-*D*_6_) δ 11.32 (s, 1H), 7.76 (s, 1H), 7.54 (s, 1H), 7.01-6.99 (m, 1H), 6.56 (d, *J* = 1.8 Hz, 1H), 3.76 (s, 3H), 3.63 (s, 4H), 3.06-3.00 (m, 4H), 1.62 (t, *J* = 6.0 Hz, 4H), 1.51-1.44 (m, 4H). ^13^C NMR (100 MHz, DMSO-*d*_6_) δ 161.69, 159.31, 158.99, 158.68, 158.37, 136.21, 127.00, 125.20, 122.17, 119.19, 117.56, 117.33, 116.46, 116.21, 109.51, 38.90, 35.30, 31.95, 29.86. HR-MS (ESI): Calcd. C_18_H_25_N_5_O, [M + H]^+^ m/z: 328.21319, found: 328.21335. HPLC retention time = 4.052 min, purity = 95.22% (λ = 254 nm).

(4-(1-Methyl-1H-pyrazol-4-yl)-1H-pyrrol-2-yl)(2,7-diazaspiro[3.5]nonan-2-yl)methanone (W-20). yellow oily substance; yield, 56.3%; ^1^H NMR (400 MHz, DMSO-*d*_6_) δ 11.44 (s, 1H), 7.77 (s, 1H), 7.54 (s, 1H), 7.06-7.03 (m, 1H), 6.59 (s, 1H), 4.15 (s, 2H), 3.76 (s, 5H), 3.12 (d, *J* = 5.0 Hz, 1H), 3.03 (s, 4H), 1.88 (d, *J* = 8.7 Hz, 4H). ^13^C NMR (100 MHz, DMSO-*d*_6_) δ 161.98, 159.89, 159.57, 159.25, 158.94, 136.15, 127.06, 124.71, 122.03, 119.06, 118.32, 117.36, 117.23, 116.09, 113.13, 40.97, 38.79, 33.08, 31.70. HR-MS (ESI): Calcd. C_16_H_21_N_5_O, [M + H]^+^ m/z: 300.18189, found: 300.18195. HPLC retention time = 3.700 min, purity = 96.07% (λ = 254 nm).

Dissolve reactant 6 (200.00 mg, 1.05 mmol) in DMF (5 mL). Add HATU (596.63 mg, 1.57 mmol) and DIPEA (405.61 mg, 3.14 mmol), and stir the mixture at room temperature for 20 minutes under a nitrogen atmosphere. Subsequently, add Secondary amine (1.57 mmol) and continue stirring at room temperature for 6 hours under a nitrogen atmosphere. Upon completion of the reaction, extract the mixture with water and ethyl acetate (25 mL × 3). Dry the organic phase over anhydrous magnesium sulfate, concentrate the filtrate, and purify the residue by column chromatography (dichloromethane:methanol = 15:1) to afford the target compound W-11, W-13~W-15, W-17, W-18, 21.

(4-(1-Methyl-1H-pyrazol-4-yl)-1H-pyrrol-2-yl)(4-methylpiperazin-1-yl)methanone (W-11). white solid; mp 222.7-224.1 ℃; yield, 20.9%. ^1^H NMR (400 MHz, DMSO-*d*_6_) δ 11.34 (s, 1H), 7.77 (s, 1H), 7.55 (s, 1H), 7.02 (dd, *J* = 2.9, 1.5 Hz, 1H), 6.62-6.58 (m, 1H), 3.76 (s, 3H), 3.71-3.63 (m, 4H), 2.36 (t, *J* = 5.2 Hz, 4H), 2.21 (s, 3H). ^13^C NMR (100 MHz, DMSO-*d*_6_) δ 161.75, 136.27, 127.01, 124.94, 117.76, 117.30, 116.58, 109.88, 55.12, 45.98, 38.93. HR-MS (ESI): Calcd. C_14_H_19_N_5_O, [M + H]^+^ m/z: 274.16624, found: 274.16653. HPLC retention time = 4.154 min, purity = 98.67% (λ = 254 nm).

(4-(1-methyl-1H-pyrazol-4-yl)-1H-pyrrol-2-yl)(4-(4-(methylsulfonyl)phenyl)piperazin-1-yl)methanone (W-13). white solid; mp 237.3-238.7 ℃; yield, 46.2%; ^1^H NMR (400 MHz, DMSO-*d*_6_) δ 11.41 (s, 1H), 7.78 (s, 1H), 7.67 (d, *J* = 9.2 Hz, 2H), 7.57 (s, 1H), 7.04 (d, *J* = 9.2 Hz, 3H), 6.70-6.67 (m, 1H), 3.84 (s, 4H), 3.77 (s, 3H), 3.47-3.42 (m, 4H), 3.06 (s, 3H). ^13^C NMR (100 MHz, DMSO-*d*_6_) δ 161.90, 153.91, 136.28, 129.15, 129.00, 127.01, 124.87, 118.04, 117.30, 116.73, 113.90, 110.15, 46.96, 44.78, 38.96. HR-MS (ESI): Calcd. C_20_H_23_N_5_O_3_S, [M + H]^+^ m/z: 414.15944, found: 414.15983. HPLC retention time = 4.178 min, purity = 98.72% (λ = 254 nm).

(4-(1-Methyl-1H-pyrazol-4-yl)-1H-pyrrol-2-yl)(morpholino)methanone (W-14). yellow solid; mp 264.9-266.1 ℃; yield, 18.4%. ^1^H NMR (400 MHz, DMSO-*d*_6_) δ 11.39 (s, 1H), 7.76 (s, 1H), 7.56 (s, 1H), 7.04 (dd, *J* = 2.9, 1.5 Hz, 1H), 6.65-6.62 (m, 1H), 3.76 (s, 3H), 3.66 (d, *J* = 4.7 Hz, 4H), 3.59 (t, *J* = 4.8 Hz, 4H). ^13^C NMR (100 MHz, DMSO-*d*_6_) δ 161.88, 136.27, 126.99, 124.75, 117.88, 117.30, 116.65, 110.01, 66.80, 38.94. HR-MS (ESI): Calcd. C_13_H_16_N_4_O_2_, [M + H]^+^ m/z: 261.13460, found: 261.13497. HPLC retention time = 4.617 min, purity = 95.59% (λ = 254 nm).

(4-(1-Methyl-1H-pyrazol-4-yl)-1H-pyrrol-2-yl)(piperidin-1-yl)methanone (W-15). yellow solid; mp 282.5-284.3 ℃; yield, 22.2%; ^1^H NMR (400 MHz, DMSO-*d*_6_) δ 11.29 (s, 1H), 7.77 (s, 1H), 7.55 (s, 1H), 6.99 (d, *J* = 1.5 Hz, 1H), 6.54 (dd, *J* = 2.7, 1.6 Hz, 1H), 3.76 (s, 3H), 3.61 (t, *J* = 5.8 Hz, 4H), 1.60 (q, *J* = 6.5 Hz, 2H), 1.45-1.52 (m, 4H). ^13^C NMR (100 MHz, DMSO-*d*_6_) δ 161.68, 136.27, 126.99, 125.36, 117.40, 117.38, 116.42, 109.33, 38.92, 26.40, 24.76. HR-MS (ESI): Calcd. C_14_H_18_N_4_O, [M + H]^+^ m/z: 259.15534, found: 259.15568. HPLC retention time = 4.149 min, purity = 96.61% (λ = 254 nm).

(1,1-Dioxidothiomorpholino)(4-(1-methyl-1H-pyrazol-4-yl)-1H-pyrrol-2-yl)methanone (W-17). yellow solid; mp 282.5-284.3 ℃; yield, 21.7%; ^1^H NMR (400 MHz, DMSO-*d*_6_) δ 11.44 (s, 1H), 7.77 (s, 1H), 7.57 (s, 1H), 7.08 (dd, *J* = 2.9, 1.5 Hz, 1H), 6.70-6.68 (m, 1H), 4.04 (s, 4H), 3.77 (s, 3H), 3.24-3.19 (m, 4H). ^13^C NMR (100 MHz, DMSO-*d*_6_) δ 162.03, 136.31, 127.06, 124.01, 118.56, 117.13, 116.86, 110.48, 51.82, 38.95. HR-MS (ESI): Calcd. C_13_H_16_N_4_O_3_S, [M + H]^+^ m/z: 309.10159, found: 309.10177. HPLC retention time = 4.028 min, purity = 97.41% (λ = 254 nm).

(4-(1-Methyl-1H-pyrazol-4-yl)-1H-pyrrol-2-yl)(pyrrolidin-1-yl)methanone (W-18). yellow solid; mp 183.3-184.6 ℃; yield, 15.7%; ^1^H NMR (400 MHz, DMSO-*d*_6_) δ 11.31 (s, 1H), 7.78 (s, 1H), 7.57 (s, 1H), 7.02 (dd, *J* = 3.1, 1.5 Hz, 1H), 6.71 (dd, *J* = 2.8, 1.5 Hz, 1H), 3.76 (s, 3H), 3.67 (s, 2H), 3.44 (s, 2H), 1.85 (d, *J* = 52.6 Hz, 4H). ^13^C NMR (100 MHz, DMSO-*d*_6_) δ 160.21, 136.24, 126.96, 126.53, 117.87, 117.49, 117.01, 109.90, 48.13, 47.21, 38.93, 26.73, 24.01. HR-MS (ESI): Calcd. C_13_H_16_N_4_O, [M + H]^+^ m/z: 245.13969, found: 245.13983. HPLC retention time = 4.103 min, purity = 96.47% (λ = 254 nm).

*N*-(1-benzylpiperidin-4-yl)-4-(1-methyl-1H-pyrazol-4-yl)-1H-pyrrole-2-carboxamide (W-21). yellow solid; mp 198.9-200.4 ℃; yield, 48.6%; ^1^H NMR (400 MHz, DMSO-*d*_6_) δ 11.31 (s, 1H), 7.73 (d, *J* = 7.0 Hz, 1H), 7.69 (s, 1H), 7.48 (s, 1H), 7.33-7.18 (m, 5H), 6.97 (d, *J* = 2.9 Hz, 1H), 6.85 (d, *J* = 1.7 Hz, 1H), 3.77 (s, 3H), 3.68 (s, 1H), 3.43 (s, 2H), 2.76 (s, 2H), 1.96 (d, *J* = 12.1 Hz, 2H), 1.71 (s, 2H), 1.50 (d, *J* = 10.2 Hz, 2H). ^13^C NMR (100 MHz, DMSO-*d*_6_) δ 160.36, 136.06, 129.36, 128.73, 127.49, 127.14, 126.74, 117.84, 117.54, 116.54, 108.02, 62.51, 52.73, 46.56, 38.97, 32.21. HR-MS (ESI): Calcd. C_21_H_25_N_5_O, [M + H]^+^ m/z: 364.21319, found: 364.21375. HPLC retention time = 4.382 min, purity = 98.47% (λ = 254 nm).

^1^H NMR and ^13^C NMR spectra of compounds

^1^H NMR spectra of compound 2

^13^C NMR spectra of compound 2

^1^H NMR spectra of compound 3

^13^C NMR spectra of compound 3

^1^H NMR spectra of compound 4

^13^C NMR spectra of compound 4

^1^H NMR spectra of compound 5

^13^C NMR spectra of compound 5

^1^H NMR spectra of compound 6

^13^C NMR spectra of compound 6

^1^H NMR spectra of compound W-01

^13^C NMR spectra of compound W-01

^1^H NMR spectra of compound W-02

^13^C NMR spectra of compound W-02

^1^H NMR spectra of compound W-03

^13^C NMR spectra of compound W-03

^1^H NMR spectra of compound W-04

^13^C NMR spectra of compound W-04

^1^H NMR spectra of compound W-05

^13^C NMR spectra of compound W-05

^1^H NMR spectra of compound W-06

^13^C NMR spectra of compound W-06

^1^H NMR spectra of compound W-07

^13^C NMR spectra of compound W-07

^1^H NMR spectra of compound W-08

^13^C NMR spectra of compound W-08

^1^H NMR spectra of compound W-09

^13^C NMR spectra of compound W-09

^1^H NMR spectra of compound W-10

^13^C NMR spectra of compound W-10

^1^H NMR spectra of compound W-11

^13^C NMR spectra of compound W-11

^1^H NMR spectra of compound W-12

^13^C NMR spectra of compound W-12

^1^H NMR spectra of compound W-13

^13^C NMR spectra of compound W-13

^1^H NMR spectra of compound W-14

^13^C NMR spectra of compound W-14

^1^H NMR spectra of compound W-15

^13^C NMR spectra of compound W-15

^1^H NMR spectra of compound W-16-boc

^13^C NMR spectra of compound W-16-boc

^1^H NMR spectra of compound W-16

^13^C NMR spectra of compound W-16

^1^H NMR spectra of compound W-17

^13^C NMR spectra of compound W-17

^1^H NMR spectra of compound W-18

^13^C NMR spectra of compound W-18

^1^H NMR spectra of compound W-19-boc

^13^C NMR spectra of compound W-19-boc

^1^H NMR spectra of compound W-19

^13^C NMR spectra of compound W-19

^1^H NMR spectra of compound W-20-boc

^13^C NMR spectra of compound W-20-boc

^1^H NMR spectra of compound W-20

^13^C NMR spectra of compound W-20

^1^H NMR spectra of compound W-21

^13^C NMR spectra of compound W-21

HRMS spectra of target compounds

HRMS spectrum of compound W-01

HRMS spectrum of compound W-02

HRMS spectrum of compound W-03

HRMS spectrum of compound W-04

HRMS spectrum of compound W-05

HRMS spectrum of compound W-06

HRMS spectrum of compound W-07

HRMS spectrum of compound W-08

HRMS spectrum of compound W-09

HRMS spectrum of compound W-10

HRMS spectrum of compound W-11

HRMS spectrum of compound W-12

HRMS spectrum of compound W-13

HRMS spectrum of compound W-14

HRMS spectrum of compound W-15

HRMS spectrum of compound W-16

HRMS spectrum of compound W-17

HRMS spectrum of compound W-18

HRMS spectrum of compound W-19

HRMS spectrum of compound W-20

HRMS spectrum of compound W-21

HPLC spectra of target compounds

| Instrument | Agilent LC1260 | Column | Agilent ZORBAX 883975-902 HPLC column (5 μm, 150 mm × 4.6 mm) |
| --- | --- | --- | --- |
| Mobile Phase | Phase A: Water;  Phase B: Methanol; A/B = 90/10, v/v | Flow Rate | 1.0 mL/min |
| Ret. Time (min) | 4.638 | Purity (%) | 98.15 |

HPLC spectrum of compound W-01

| Instrument | Agilent LC1260 | Column | Agilent ZORBAX 883975-902 HPLC column (5 μm, 150 mm × 4.6 mm) |
| --- | --- | --- | --- |
| Mobile Phase | Phase A: Water;  Phase B: Methanol; A/B = 90/10, v/v | Flow Rate | 1.0 mL/min |
| Ret. Time (min) | 4.351 | Purity (%) | 96.48 |

HPLC spectrum of compound W-02

| Instrument | Agilent LC1260 | Column | Agilent ZORBAX 883975-902 HPLC column (5 μm, 150 mm × 4.6 mm) |
| --- | --- | --- | --- |
| Mobile Phase | Phase A: Water;  Phase B: Methanol; A/B = 90/10, v/v | Flow Rate | 1.0 mL/min |
| Ret. Time (min) | 4.149 | Purity (%) | 99.01 |

HPLC spectrum of compound W-03

| Instrument | Agilent LC1260 | Column | Agilent ZORBAX 883975-902 HPLC column (5 μm, 150 mm × 4.6 mm) |
| --- | --- | --- | --- |
| Mobile Phase | Phase A: Water;  Phase B: Methanol; A/B = 90/10, v/v | Flow Rate | 1.0 mL/min |
| Ret. Time (min) | 4.193 | Purity (%) | 99.54 |

HPLC spectrum of compound W-04

| Instrument | Agilent LC1260 | Column | Agilent ZORBAX 883975-902 HPLC column (5 μm, 150 mm × 4.6 mm) |
| --- | --- | --- | --- |
| Mobile Phase | Phase A: Water;  Phase B: Methanol; A/B = 90/10, v/v | Flow Rate | 1.0 mL/min |
| Ret. Time (min) | 4.086 | Purity (%) | 95.01 |

HPLC spectrum of compound W-05

| Instrument | Agilent LC1260 | Column | Agilent ZORBAX 883975-902 HPLC column (5 μm, 150 mm × 4.6 mm) |
| --- | --- | --- | --- |
| Mobile Phase | Phase A: Water;  Phase B: Methanol; A/B = 90/10, v/v | Flow Rate | 1.0 mL/min |
| Ret. Time (min) | 3.996 | Purity (%) | 98.13 |

HPLC spectrum of compound W-06

| Instrument | Agilent LC1260 | Column | Agilent ZORBAX 883975-902 HPLC column (5 μm, 150 mm × 4.6 mm) |
| --- | --- | --- | --- |
| Mobile Phase | Phase A: Water;  Phase B: Methanol; A/B = 90/10, v/v | Flow Rate | 1.0 mL/min |
| Ret. Time (min) | 4.157 | Purity (%) | 97.39 |

HPLC spectrum of compound W-07

| Instrument | Agilent LC1260 | Column | Agilent ZORBAX 883975-902 HPLC column (5 μm, 150 mm × 4.6 mm) |
| --- | --- | --- | --- |
| Mobile Phase | Phase A: Water;  Phase B: Methanol; A/B = 90/10, v/v | Flow Rate | 1.0 mL/min |
| Ret. Time (min) | 4.129 | Purity (%) | 99.05 |

HPLC spectrum of compound W-08

| Instrument | Agilent LC1260 | Column | Agilent ZORBAX 883975-902 HPLC column (5 μm, 150 mm × 4.6 mm) |
| --- | --- | --- | --- |
| Mobile Phase | Phase A: Water;  Phase B: Methanol; A/B = 90/10, v/v | Flow Rate | 1.0 mL/min |
| Ret. Time (min) | 4.170 | Purity (%) | 100.00 |

HPLC spectrum of compound W-09

| Instrument | Agilent LC1260 | Column | Agilent ZORBAX 883975-902 HPLC column (5 μm, 150 mm × 4.6 mm) |
| --- | --- | --- | --- |
| Mobile Phase | Phase A: Water;  Phase B: Methanol; A/B = 90/10, v/v | Flow Rate | 1.0 mL/min |
| Ret. Time (min) | 4.144 | Purity (%) | 96.42 |

HPLC spectrum of compound W-10

| Instrument | Agilent LC1260 | Column | Agilent ZORBAX 883975-902 HPLC column (5 μm, 150 mm × 4.6 mm) |
| --- | --- | --- | --- |
| Mobile Phase | Phase A: Water;  Phase B: Methanol; A/B = 90/10, v/v | Flow Rate | 1.0 mL/min |
| Ret. Time (min) | 4.154 | Purity (%) | 98.67 |

HPLC spectrum of compound W-11

| Instrument | Agilent LC1260 | Column | Agilent ZORBAX 883975-902 HPLC column (5 μm, 150 mm × 4.6 mm) |
| --- | --- | --- | --- |
| Mobile Phase | Phase A: Water;  Phase B: Methanol; A/B = 90/10, v/v | Flow Rate | 1.0 mL/min |
| Ret. Time (min) | 3.998 | Purity (%) | 99.35 |

HPLC spectrum of compound W-12

| Instrument | Agilent LC1260 | Column | Agilent ZORBAX 883975-902 HPLC column (5 μm, 150 mm × 4.6 mm) |
| --- | --- | --- | --- |
| Mobile Phase | Phase A: Water;  Phase B: Methanol; A/B = 90/10, v/v | Flow Rate | 1.0 mL/min |
| Ret. Time (min) | 4.178 | Purity (%) | 98.72 |

HPLC spectrum of compound W-13

| Instrument | Agilent LC1260 | Column | Agilent ZORBAX 883975-902 HPLC column (5 μm, 150 mm × 4.6 mm) |
| --- | --- | --- | --- |
| Mobile Phase | Phase A: Water;  Phase B: Methanol; A/B = 90/10, v/v | Flow Rate | 1.0 mL/min |
| Ret. Time (min) | 4.617 | Purity (%) | 95.59 |

HPLC spectrum of compound W-14

| Instrument | Agilent LC1260 | Column | Agilent ZORBAX 883975-902 HPLC column (5 μm, 150 mm × 4.6 mm) |
| --- | --- | --- | --- |
| Mobile Phase | Phase A: Water;  Phase B: Methanol; A/B = 90/10, v/v | Flow Rate | 1.0 mL/min |
| Ret. Time (min) | 4.149 | Purity (%) | 96.61 |

HPLC spectrum of compound W-15

| Instrument | Agilent LC1260 | Column | Agilent ZORBAX 883975-902 HPLC column (5 μm, 150 mm × 4.6 mm) |
| --- | --- | --- | --- |
| Mobile Phase | Phase A: Water;  Phase B: Methanol; A/B = 90/10, v/v | Flow Rate | 1.0 mL/min |
| Ret. Time (min) | 4.451 | Purity (%) | 96.10 |

HPLC spectrum of compound W-16

| Instrument | Agilent LC1260 | Column | Agilent ZORBAX 883975-902 HPLC column (5 μm, 150 mm × 4.6 mm) |
| --- | --- | --- | --- |
| Mobile Phase | Phase A: Water;  Phase B: Methanol; A/B = 90/10, v/v | Flow Rate | 1.0 mL/min |
| Ret. Time (min) | 4.028 | Purity (%) | 97.41 |

HPLC spectrum of compound W-17

| Instrument | Agilent LC1260 | Column | Agilent ZORBAX 883975-902 HPLC column (5 μm, 150 mm × 4.6 mm) |
| --- | --- | --- | --- |
| Mobile Phase | Phase A: Water;  Phase B: Methanol; A/B = 90/10, v/v | Flow Rate | 1.0 mL/min |
| Ret. Time (min) | 4.103 | Purity (%) | 96.47 |

HPLC spectrum of compound W-18

| Instrument | Agilent LC1260 | Column | Agilent ZORBAX 883975-902 HPLC column (5 μm, 150 mm × 4.6 mm) |
| --- | --- | --- | --- |
| Mobile Phase | Phase A: Water;  Phase B: Methanol; A/B = 90/10, v/v | Flow Rate | 1.0 mL/min |
| Ret. Time (min) | 4.052 | Purity (%) | 95.22 |

HPLC spectrum of compound W-19

| Instrument | Agilent LC1260 | Column | Agilent ZORBAX 883975-902 HPLC column (5 μm, 150 mm × 4.6 mm) |
| --- | --- | --- | --- |
| Mobile Phase | Phase A: Water;  Phase B: Methanol; A/B = 90/10, v/v | Flow Rate | 1.0 mL/min |
| Ret. Time (min) | 3.700 | Purity (%) | 96.07 |

HPLC spectrum of compound W-20

| Instrument | Agilent LC1260 | Column | Agilent ZORBAX 883975-902 HPLC column (5 μm, 150 mm × 4.6 mm) |
| --- | --- | --- | --- |
| Mobile Phase | Phase A: Water;  Phase B: Methanol; A/B = 90/10, v/v | Flow Rate | 1.0 mL/min |
| Ret. Time (min) | 4.382 | Purity (%) | 98.47 |

HPLC spectrum of compound W-21

**Fig. S1** Original immunoblot data in Fig 1D.

**Fig. S2** Original immunoblot data in Fig 4B-4C, 4E-H.

**Fig. S3** Original immunoblot data in Fig 5G.
